# Advanced Switchable Molecules and Materials for Oil Recovery and Oily Waste Cleanup

**DOI:** 10.1002/advs.202004082

**Published:** 2021-05-27

**Authors:** Yi Lu, Yeling Zhu, Fan Yang, Zhenghe Xu, Qingxia Liu

**Affiliations:** ^1^ Department of Chemical and Materials Engineering University of Alberta Edmonton Alberta T6G 1H9 Canada; ^2^ College of New Materials and New Energies Shenzhen Technology University Shenzhen 518118 P. R. China; ^3^ Department of Materials Science and Engineering Southern University of Science and Technology Shenzhen 518055 P. R. China

**Keywords:** magnetic‐responsive materials, oil recovery, oily waste cleanup, stimuli‐responsive materials, switchable molecules, switchable materials

## Abstract

Advanced switchable molecules and materials have shown great potential in numerous applications. These novel materials can express different states of physicochemical properties as controlled by a designated stimulus, such that the processing condition can always be maintained in an optimized manner for improved efficiency and sustainability throughout the whole process. Herein, the recent advances in switchable molecules/materials in oil recovery and oily waste cleanup are reviewed. Oil recovery and oily waste cleanup are of critical importance to the industry and environment. Switchable materials can be designed with various types of switchable properties, including i) switchable interfacial activity, ii) switchable viscosity, iii) switchable solvent, and iv) switchable wettability. The materials can then be deployed into the most suitable applications according to the process requirements. An in‐depth discussion about the fundamental basis of the design considerations is provided for each type of switchable material, followed by details about their performances and challenges in the applications. Finally, an outlook for the development of next‐generation switchable molecules/materials is discussed.

## Graphical abstract

Switchable molecules and materials have been drawing increasing interests in the oil recovery and oily wastes cleanup to improve chemical/energy efficiency and process sustainability. Herein, a comprehensive review is provided to introduce the fundamental basis for the design and fabrication of advanced switchable materials, to present their recent progress in applications, and to discuss their limitations and future outlooks.

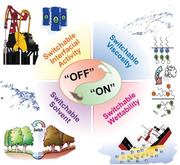

## 1 Introduction

### 1.1 Motivations in Oil Recovery Applications

**Scheme 1:**
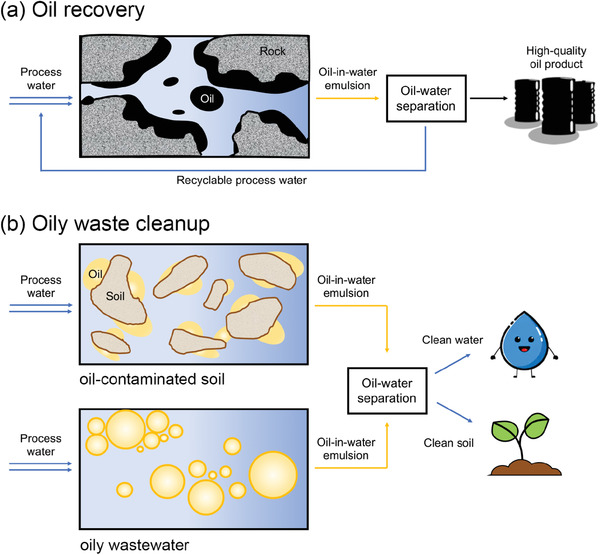
a) Schematic illustration of a typical water flooding oil recovery process. b) Schematic illustrations of several oily waste treatments. Top: Remediation of oil‐contaminated soil. Bottom: Remediation of oily wastewater.

Recovering oil from natural resources is one of the essential activities in human society. Currently, oil recovery is mostly referred to as the extraction of petroleum hydrocarbons that exist in various forms, including light oil, heavy crude, offshore oil reserves, kerogen‐bearing shales, and oil sands, despite that recently there are also trends toward bio‐oil production from biomass. **Scheme** **1**a shows a classic process of water‐flooding enhanced oil recovery (EOR) process. Typically, the oil recovery process involves multiple steps, with various parameters that have to be carefully controlled. The objective of modulating operating parameters is to maintain an optimum condition for oil production while at the same time reducing chemical/energy consumption. However, it has been long and widely recognized that some specific conditions favoring one operation stage could become detrimental to the downstream operations during the recovery process. For example, a lower oil‐water interfacial tension (IFT) assists in releasing the crude oil from the host solids in a water‐based oil sands extraction process. On the other hand, in the subsequent stage of oil‐water separation, the low oil‐water IFT may cause the generation of stable oil‐in‐water (O/W) emulsions, making valuable oil products too difficult to be reclaimed.^[^ ^1^ ^]^ Similarly, displacing fluids with higher viscosity improves the mobility of heavy oils in underground porous solids and thus benefits the EOR.^[^ ^2^ ^]^ Unfortunately, the injection of such highly viscous fluids requires high pump‐in and pump‐out pressures, which substantially increases the difficulty and cost of operations. Besides, solvents that could dissolve and extract more hydrocarbons are much harder to recycle from crude oil products, causing solvent losses.^[^ ^3^ ^]^ Traditional industrial solutions to these dilemmas can be briefed as using one specific chemical for one specific task. For example, surfactants are applied to reduce oil‐water IFT, followed by adding demulsifiers to get rid of water from products. However, the deployment of single‐functional processing aids increases the consumption of chemicals, which is considered to be both inefficient and costly.

### 1.2 Motivations in Oily Wastewater Cleanup Applications

With the rapid growth of the modern industry, there are also emerging needs to clean up oily wastes that can be generated during the industrial operations or by accidents. For example, it is almost inevitable that petroleum hydrocarbons will contaminate the process water used for oil recovery. These hydrocarbon‐containing oily wastes should be carefully treated before being discharged. Moreover, oil‐spill accidents have been frequently reported during oil exploration, drilling, transportation, and storage processes, which may contaminate soil resources. The occurrence of these accidents can have a catastrophic impact on public health and the environment.^[^ ^4^ ^]^ Currently, numerous efforts are being made to remediate the oil‐contaminated water and/or soil bodies in order to improve the eco‐sustainability of the environments surrounding these sites,^[^ ^5^ ^]^ as illustrated in Scheme 1b. Most of the current techniques for the oily waste cleanup are transferred from the oil recovery process either directly or indirectly. However, it is still worth mentioning that the environmental treatment process is focused more on the recovery of clean water/soil rather than on producing high‐quality oil. The quality of the water/soil is the major criteria to assess the efficiency of the oily waste cleanup processes. Novel materials have to be carefully assessed by their effectiveness at each stage of the operation and their potential effects on the ecosystems.

### 1.3 “Smart” Solutions: Switchable Molecules and Materials

Recent advances in switchable molecules and materials provide a new direction for developing novel multi‐functional materials for oil recovery and oily waste cleanup applications. Switchable materials are capable of expressing different states of physicochemical properties as controlled by a well‐defined stimulus. Such switchability enables these advanced materials to accomplish more than one task in operations. For example, switchable surfactants could be introduced in their interfacial‐active state to reduce the IFT and enhance the miscibility of oil in water. Subsequently, the same surfactant could be switched to its interfacial‐inactive state to facilitate the oil‐water separation stage.^[^ ^6^ ^]^ Such an “intelligent” process could be triggered by a designated external stimulus, e.g., temperature,^[^ ^7^ ^]^ pH,^[^ ^8^ ^]^ UV radiation,^[^ ^9^ ^]^ CO_2_/N_2_ gas.^[^ ^6^, ^10^ ^]^ Since the switching behaviors are typically activated through the responsiveness of the materials to a particular stimulus, switchable materials are also commonly known as the “responsive materials” and named after their triggers, e.g., thermal‐responsive materials and CO_2_‐responsive surfactants.

However, the implementation of switchable molecules and materials in the current industry is still a challenging task. Firstly, the use of switchable materials could be challenged by the system complexity, both for the oil recovery process and for the oily waste cleanup treatment. The oil phase in these application scenarios is known to contain various natural compositions, which makes it readily complicated in chemistry. Besides, the system complexity may also arise from various processing conditions, including water chemistry, solid compositions, geological conditions, and biological environments. The successful implementation of switchable materials into such systems demands a comprehensive understanding of the interactions between each component. Secondly, it is also essential to select the most appropriate switching trigger based on the conditions of the actual application. For example, the strategy of applying UV light as the switching trigger is suitable for applications with good water transparency, whereas thermo‐responsive materials are sometimes chosen if the operation has readily involved a significant temperature gradient (e.g., in situ EOR at hot reservoirs).^[^ ^11^ ^]^ Meantime, CO_2_ gas is also commonly used as a switching trigger, since it is largely available, easy‐to‐apply, and reasonably priced.^[^ ^6^, ^10^ ^]^ Last but not least, switchable materials are typically more difficult and costly to synthesize. Thus, industrial applications must balance between the investment in switchable materials and their economic efficiency. Fortunately, many switchable materials with reversible switching behaviors have the potential to be fully or partially recycled with the proper design of the operation process, which could largely reduce their corresponding cost.

Apart from the switchable molecules and materials, this review also covers the recent advances in magnetic‐responsive materials, which can be considered as a special type of stimuli‐responsive material. Magnetic‐responsive materials do not usually switch their physiochemical properties, but instead, have their motion and pattern of movement respond to an externally applied magnetic field. This type of responsiveness is especially meaningful to the collection and micro‐transportation of materials. Since magnetic‐responsive materials are also of great interest in both scientific exploration and industrial applications, we have included this special type of advanced material in this review for the completeness of our discussion.

In general, switchable/responsive materials have the ability to perform multiple roles. Their successful implementations would significantly reduce the dosage of chemical additions/energy input, improve process sustainability, and alleviate environmental concerns. It is not surprising to see that advanced switchable materials have been considered as the frontier technology in a variety of fields, including oil recovery, oily waste cleanup, drug delivery,^[^ ^12^ ^]^ sensors,^[^ ^13^ ^]^ catalyst,^[^ ^14^ ^]^ tissue engineering,^[^ ^15^ ^]^ cosmetics,^[^ ^16^ ^]^ and food industry.^[^ ^17^ ^]^

In this review, we present state‐of‐the‐art studies on advanced switchable molecules and materials in oil recovery and oily waste cleanup applications. The applications of these advanced materials are classified into four categories based on their switchable property: switchable interfacial activity (Section 2), switching viscosity (Section 3), switchable solvent (Section 4), and switchable wettability (Section 5). It has to be mentioned that the primary frame of this article is arranged by the types of the switchable physiochemical property (e.g., switchable interfacial activity), instead of the type of the switching triggers (e.g., temperature, pH, CO_2_). We believe that such an organization provides insights into the connection between the current scientific advances in switchable materials and the emerging requirements in real applications. Therefore, in each section, we will briefly introduce the fundamental knowledge involved in the industrial process, before explaining the technical issues that are difficult to be resolved with conventional materials. After that, we will present novel switchable molecules and materials used in oil recovery and oily waste cleanup applications by discussing their performances and challenges. At the end of this review, the future outlook for the development of next‐generation switchable materials is discussed (Section 6).

## 2 Switchable Interfacial Activity

### 2.1 Role of Interfacial Activity

Interfacial activity is of critical importance to both oil recovery and oily waste cleanup applications. The interfacial‐active materials could efficiently decrease the oil‐water interfacial tension (IFT), reduce the capillary pressure in porous media, and/or alter the wettability of solids in reservoir. A desired interfacial activity can be generated by the contribution of surface‐active natural surfactants connately existed in the crude oils,^[^ ^1^, ^18^ ^]^ e.g., naphthenic acids, asphaltenes, and resins, or by the externally deployed amphiphilic materials typically in the form of surfactants,^[^ ^19^ ^]^ polymers,^[^ ^20^ ^]^ or particles.^[^ ^21^ ^]^

The role of interfacial activity is complex and can be paradoxical in different stages of the operation. Tuning interfacial activities can be favorable for certain subprocesses but detrimental to others. Masliyah et al. showed that decreasing oil‐water IFT (*γ*_ow_) is thermodynamically essential to reduce the free energy change (Δ*G*) for the oils to detach from host solids^[^ ^22^ ^]^

(1) $$\Delta G=\Delta A\cdot \gamma_{\mathrm{ow}}\left(1-\cos\theta \right)$$

where Δ*A* is the area change of an oil‐solid interface being replaced by an oil‐water interface, and *θ* is the contact angle through the aqueous phase. It is much easier for the oil detachment to occur when the energy barrier is low. Besides, a lower value of *γ*_ow_ could reduce the capillary pressure (*P*_c_) between the immiscible phases, as illustrated by the Young‐Laplace equation^[^ ^23^ ^]^

(2) $$P_{c}=\frac{2\gamma_{\mathrm{ow}}\cos\theta }{r_{c}}$$

where *r*_c_ is the effective radius of the interface. Decreasing the capillary pressure allows the washing fluids to carry out more oils trapped by the porous media, especially those in the capillary pores (ultra‐low *r*_c_ values). On the other hand, it is also well‐known that the low oil‐water IFT is the primary reason for the formation of undesired emulsions.^[^ ^1^, ^22^ ^]^ These stable emulsions could contain a considerable amount of heavy oil product that may ended in the oily wastewater.

Typical strategies to alleviate the side effects of introducing the interfacial activity, especially the unwanted formation of ultra‐stable O/W emulsions, involve the optimization of the chemical dosage, the addition of a secondary processing aid (e.g., demulsifier), or the application of physical forces (e.g., centrifugation or electrical field). However, these traditional methods either sacrifice subprocess efficiency, accumulate potential pollutants, or require energy‐intensive equipment. Interfacial‐active responsive materials that feature switchable interfacial activity are promising alternatives to overcome technical difficulties. The point of introducing switchable interfacial‐active materials is to precisely modulate the interfacial activity that best fits for each subprocess and thereby achieves an overall optimized performance with fewer chemical additions.

### 2.2 Switchable Surfactants

#### 2.2.1 Switchable Surfactants for Oil Recovery

**Figure 1:**
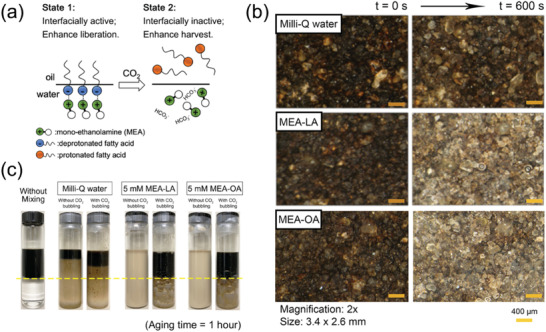
a) Proposal concept of CO_2_‐responsive surfactants in heavy oil recovery applications and their switching mechanism. b) CO_2_‐responsive surfactants can enhance heavy oil liberation based on the visual observations. The surfaces of petroleum ores became “brighter” after being treated by CO_2_‐responsive surfactants, which means that more heavy oil was liberated. c) CO_2_‐responsive surfactants can also enhance heavy oil harvest after the triggering of CO_2_ response. The heavy oil‐in‐water emulsions was very stable in the presence of CO_2_‐responsive surfactants, but was also able to achieve fast and near‐complete phase separation after CO_2_ bubbling. b,c) Reproduced with permission.^[^ ^6^ ^]^ Copyright 2020, American Institute of Chemical Engineers.

Surfactants are widely used to reduce oil‐water interfacial tension for enhanced oil recovery. Conventional surfactants are known to improve the liberation of heavy oil from their host rock/solids, but often suffer from the detrimental effects of reduced harvest efficiency and an increased amount of oily tailings.^[^ ^19^ ^]^ On the other hand, switchable surfactants are proposed to enhance both the heavy oil liberation and harvest by utilizing their switchable interfacial activity. Lu et al. reported CO_2_‐responsive surfactants that were easily formed by the ion pairs of mono‐ethanolamine (MEA) with long‐chain fatty acids (LCFAs) at an equal molar ratio at the oil‐water interface.^[^ ^10a^ ^]^ Bubbling of CO_2_ gas into the aqueous phase decreases the bulk pH value and thereby causes the protonation of fatty acid. Since the protonated (non‐ionic) fatty acid cannot associate with MEA^+^ and is not interfacially active itself, both compositions will leave the interface, which results in the loss of interfacial activity (**Figure** **1**a). This series of CO_2_‐responsive surfactants also features an easily tunable switching pH by applying different types of LCFAs, such that the surfactants can be customized according to the aqueous pH value of the onsite process water.^[^ ^10a^ ^]^ Later on, Lu et al.^[^ ^6^ ^]^ demonstrated that these CO_2_‐responsive surfactants significantly enhanced the release of heavy oil from host solids (Figure 1b). A subsequent stage of oil‐water separation could be achieved efficiently by bubbling CO_2_ into the emulsions. The volume of the phase‐separated heavy oil is almost identical to the oil being added originally (Figure 1c). This series of surfactants were successfully tested in a water‐based Canadian oil sands extraction process and significantly enhanced the overall bitumen recovery.^[^ ^24^, ^45^ ^]^ The same research group further developed CO_2_‐responsive surfactants with a pseudo‐Gemini structure to improve the interfacial activity,^[^ ^25^ ^]^ which shows the potentials to deliver similar performance at a lower dosage. On the other hand, switchable ethoxylated amine surfactants (C_12‐14_N(EO)_X_) were also investigated by Chen et al. for CO_2_ EOR in carbonate reservoirs.^[^ ^26^ ^]^ These surfactants can switch from the nonionic state (unprotonated) in dry CO_2_ to cationic (protonated) in the presence of an aqueous phase with a pH less than 6. The main reason of choosing switchable amine surfactants in this study is to minimize the adsorption of surfactants on the carbonate minerals.^[^ ^27^ ^]^

Switchable surfactants are also able to assist the transportation of highly viscous oils, utilizing their switchable interfacial activity. In certain industrial scenarios, the extraction products of heavy oils need to be pipelined from the extraction sites to downstream operators. It is thereby proposed that heavy crude oils could be emulsified in water by switchable surfactants, such that the as‐generated crude oil‐in‐water emulsion would have a reduced viscosity for convenient pipeline transportation. Thanks to the switching property of the switchable surfactants, the heavy oil products can eventually be released from the emulsion after transportation without affecting their quality. Based on this concept, Jessop's group evaluated a series of structurally diverse amidines with different hydrophilic or hydrophobic chains for pipelining heavy crude oil and CO_2_‐triggered demulsification.^[^ ^28^ ^]^ It was reported that certain amidines that incorporated with the indigenous surfactants in the crude oil led to the formation of stable O/W emulsions; such interfacial activity could be switched off by purging CO_2_. Similarly, Lu and his co‐workers developed CO_2_ switchable emulsions to improve the heavy oil flows in hydro‐transportation.^[^ ^10^, ^29^ ^]^ At the end of the pipelines, CO_2_ gas was bubbled to trigger the demulsification and the release of heavy oil, while the aqueous phase could be recycled. However, the primary obstacle of applying switchable surfactants in pipeline transportation includes the insufficient phase separation that exists in both the resulting oil product (organic) and the carrier fluid (aqueous) phases.^[^ ^28^ ^]^

More recently, switchable surfactants have been further explored as the key component to build up switchable microemulsions system. Microemulsions have attracted diverse interests in EOR applications due to their capability of carrying a large quantity of crude oil out of the reservoir.^[^ ^30^ ^]^ Typically, the formulation of microemulsions contains surfactants, cosurfactants, cosolvents, water, and electrolytes. The balance between the above‐mentioned species is the most critical criterion for the stabilization of microemulsions, which can be elucidated by studying the phase behaviors at different compositions. When the microemulsions are formulated by switchable surfactants, their phase behavior can be easily modulated by tuning the interfacial activity of the switchable surfactants.

**Figure 2:**
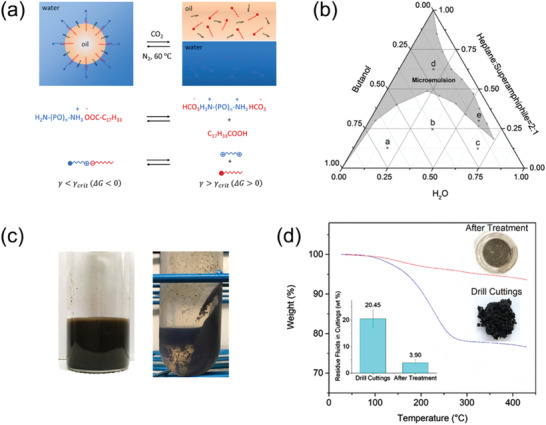
a) Schematic diagram illustrating the formation of O/W microemulsion using a superamphiphile assembled by Jeffamine D230 and oleic acid at 1:1 molar ratio, as well as the corresponding switching mechanism induced by CO_2_ bubbling. b) Pseudoternary phase diagram drawn in mass fractions for mixtures of water, 1‐butanol and superamphiphile in heptan. c) Left: Mixture of 1 g drill cuttings and 5 g O/W microemulsion (left), and Right: Oil extracted from the drill cuttings after CO_2_ purging for 20 s and centrifugation at 1000 rpm for 15 min. d) Thermal gravimetric analysis (TGA) results of the drill cuttings before and after treatment by CO_2_‐responsive microemulsions. a–d) Reproduced with permission.^[^ ^10f^ ^]^ Copyright 2018, Elsevier.

Chen et al. investigated CO_2_‐responsive O/W microemulsions using a switchable superamphiphile assembled by Jeffamine D230 and oleic acid (OA).^[^ ^10f^ ^]^ This novel microemulsion can be destabilized rapidly (within 20 s) and phase‐separated completely by CO_2_ bubbling (**Figure** **2**). It was demonstrated that a significant amount of oil (drilling fluid) could be extracted from the drill cuttings using such microemulsion system. The authors also claimed that the switchable superamphiphile could be reconstructed after purging of N_2_ gas at 60 °C, suggesting the possibility of recycling. Similarly, Brown et al. also developed CO_2_‐responsive microemulsions based on reactive ionic liquids. These microemulsions were proposed as the replacement to conventional cleaning agents and emulsifiers.^[^ ^31^ ^]^ In addition, a few more CO_2_‐switchable microemulsions have been reported for broad aspects of applications.^[^ ^32^ ^]^

Apart from the utilization of CO_2_‐induced phase inversions, the switchable microemulsion system could also be constructed by light‐responsive surfactants.^[^ ^33^ ^]^ Wolff's group reported a series of UV‐responsive microemulsion systems containing AOT (*bis*‐2‐ethylhexylsulfosuccinate sodium salt), isooctane, and water.^[^ ^33a–d^ ^]^ The authors examined a wide‐range selection of the light‐responsive surfactants and found that azobenzene‐containing microemulsion exhibited the most promising behavior due to its rapid and fully reversible phase inversion.^[^ ^33d^ ^]^ Meanwhile, Eastoe et al. reported a light‐induced microemulsion that contained UV‐sensitive Gemini surfactants for controllable encapsulation and delivery systems.^[^ ^33e^ ^]^ For the readers with further interests in the switchable microemulsions, Zhang and Feng recently summarized the state‐of‐the‐art developments in this field and their future perspectives.^[^ ^34^ ^]^

#### 2.2.2 Switchable Surfactants for Oily Waste Cleanup

Environmental regulators have been recently raising concerns that valuable soil resources are contaminated by oily wastes, such as polycyclic aromatic hydrocarbons (PAHs), polychlorinated biphenyls (PCBs), total petroleum hydrocarbons (TPH), and pharmaceuticals and personal care products (PPCPs). There are a number of possible reasons causing the contamination of soil, including uncontrolled/insufficiently controlled release of industrial wastes, pipeline leakage due to corrosion or breakdown, and accidental spills caused by storage tank failures.

Surfactant‐enhanced soil washing or soil flushing is one of the most versatile techniques for soil remediation, both of which are generally applicable to most organic contaminants from solids. Similar to the oil recovery process, surfactant‐enhanced soil washing/flushing process uses surfactant solutions to clean up the oily contaminated on soil solid surfaces. After washing/flushing, the effluents would contain a high amount of organic waste and need to be decontaminated before discharging. Typically, there are two major approaches to treat oily effluents: degradation and separation. Degradation is more appropriate for effluents with lower oil concentration, in which the organic content in the aqueous phase could be eliminated efficiently by photocatalysis,^[^ ^35^ ^]^ oxidation,^[^ ^36^ ^]^ plasma technology,^[^ ^5b^ ^]^ electrochemistry,^[^ ^37^ ^]^ or biological treatment.^[^ ^38^ ^]^ When the soil washing/flushing effluents contain a relatively higher amount of oil, oil‐water separation can create additional economic values.

**Figure 3:**
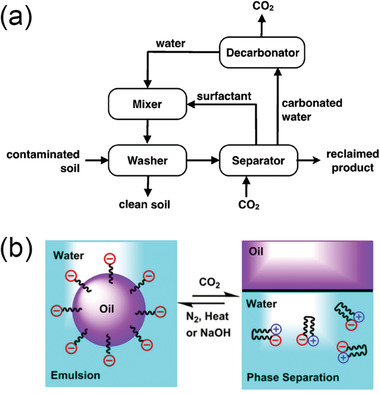
a) A proposed protocol of soil washing/flushing using CO_2_ switchable anionic surfactants. Reproduced with permission.^[^ ^39a^ ^]^ Copyright 2014, The Royal Society of Chemistry. b) Schematic illustration of the emulsification and demulsification process using CO_2_‐responsive surfactants. Reproduced with permission.^[^ ^41^ ^]^ Copyright 2018, American Chemical Society.

**Figure** **3**a shows a proposed protocol of soil washing/flushing followed by oil‐water separation. This protocol is conceptually similar to the application of switchable surfactants in the oil recovery process discussed in Section 2.2.1. However, it has to be emphasized that the primary focus in the soil remediation process is to remove the toxic oil contaminates and restore clean soil resources, whereas, in the oil recovery process, the priority is to produce high‐quality crude oil with less possible environmental impact (Scheme 1). Namely, one of the biggest challenges in the soil‐washing process is to ensure that the remediated soil solids are clean and eco‐friendly. Therefore, switchable surfactants used for soil remediation purposes must be carefully designed with properly charged moiety to avoid specific adsorption to the soil solids, which may cause surfactant accumulation in the “clean” soil and thereby secondary pollution. For example, cationic surfactants have been demonstrated to perform poorly in soil washing processes where the solid mineral surface has negative charges.^[^ ^39^ ^]^ Ceschia et al. explored various CO_2_ switchable anionic surfactants to remediate oil‐contaminated silica sand (i.e., negatively charged sand) by ex situ soil washing.^[^ ^39a^ ^]^ The switchable surfactants were capable of removing crude oil from contaminated sands with a similar performance as the commercial nonionic surfactant Triton X‐100 at both room and elevated temperature. More importantly, the oily contaminants in the effluents were easily separated out by deactivating the surface activity of switchable surfactants throughout CO_2_ purging. At the optimum condition, 97% of the crude oil contaminants were successfully removed from the sands, while the remaining oil in the washing fluid only counted for ≈5%. The residue surfactants on the sand surface and in the aqueous phase were about 0.45% and 0.48%, respectively, indicating negligible surfactant accumulation throughout cycles. Similarly, Tian et al. investigated the reversible solubility of typical PAHs in the presence of CO_2_‐switchable surfactant (2‐*n*‐lauryl‐1,1,3,3‐tetramethyl guanidine, DTMG) for their potential applications in the soil washing process.^[^ ^40^ ^]^ More recently, Xu et al. reported a novel CO_2_‐switchable anionic surfactant based on a sulfate salt surfactant (i.e., 11‐dimethylaminoundecyl sulfate sodium salt, DUSNa) for the oil‐contaminated soil remediation (Figure 3b).^[^ ^41^ ^]^ The authors claimed that the recovery ratio of mineral oil was around 92.1–94.1%, whereas the residual oil on “clean” sands only counted for 2.8–3.2% of the original amount, or less than 0.17% in the aspect of the total mass. Furthermore, the total organic content (TOC) and chemical oxygen demand (COD) in the process water were measured at less than 40 mg L^−1^ after treatment, indicating excellent decontamination of the process water as well.

The current advances of switchable surfactants in heavy oil recovery and oily waste cleanup applications are summarized in **Table** **1**. Despite the advantages of switchable surfactants in various applications, there are still challenges to be resolved, which are mainly related to the salt effect and the surfactants loss. To the best of our knowledge, switchable surfactants for these applications have mostly been designed with switchable interfacial activity triggered by CO_2_ bubbling because CO_2_ is readily available as a byproduct in many applications and inexpensive to use. Since the CO_2_ response is a subphase of the pH response, which relies on the change of molecular amphiphilicity during the protonation/deprotonation process,^[^ ^10h^ ^]^ these CO_2_‐responsive surfactants must have at least one state carrying electro‐charges, e.g., the interfacial‐active fatty acid is anionic (deprotonated). Therefore, the presence of dissolved ions in the process water, especially multivalent ions such as Ca^2+^ and Mg^2+^, could negatively impact the switchability and as a result, the performance of the responsive surfactants. For example, anionic fatty acids are known to have a strong affinity with Ca^2+^,^[^ ^42^ ^]^ resulting in insoluble precipitations and, thereby, the degradation of interfacial activity.^[^ ^25a^ ^]^ Besides, the charged state of switchable surfactants might also cause the specific adsorption onto reservoir solids due to electrostatic interaction,^[^ ^43^ ^]^ which further deteriorates the ability of surfactants to reduce oil‐water IFT. As for switchable microemulsions formulated by switchable surfactants, one of the principal limitations is their economic efficiency. The preparation of switchable microemulsions would typically consume a large amount of switchable surfactant. It should also be taken into account that the porous media can also influence the phase behavior of microemulsions,^[^ ^44^ ^]^ which might also impact the performance of switchable microemulsions during in situ operations.

**Table 1:** Recent advances of switchable surfactants in oil recovery and oily waste cleanup applications

Application	Surfactant	Switching Trigger	Performance	Ref.
Oil recovery	MEA‐LCFA	CO_2_	Heavy oil liberation: from 22.38% to 74.67%; Heavy oil harvest: ≈100% after CO_2_ bubbling.	^[^ ^6^ ^]^
			Canadian oil sands extraction by water‐based technology: from 15.0% to 50.4%	^[^ ^24b^ ^]^
			Canadian oil sands extraction by aqueous‐non‐aqueous hybrid technology: from 74.2% to 88.6%.	^[^ ^45^ ^]^
	DETA‐2LA, TETA‐2LA	CO_2_	Heavy oil liberation: from 24% to 77%; Enhanced oil‐water separation after CO_2_ bubbling.	^[^ ^25a^ ^]^
	C_12‐14_N(EO)_2_, C_12‐14_N(EO)_5_, C_12‐14_N(EO)_15_	CO_2_	Stabilize CO_2_‐in‐water foams from pH 4–6 at high temperature up to 120 °C and high salinities up to 182 g L^−1^.	^[^ ^26^ ^]^
	Jeffamine D‐230 + OA (1:1), 1‐butanol as the co‐surfactant	CO_2_	Transparent microemulsion system; Fast demulsification with CO_2_ bubbling (20 s); Reversible switchability; Recover oil from drilling cuts, with less than 4 wt% of oil left on solids.	^[^ ^10f^ ^]^
	[bmim][Triazolide]	CO_2_	Microemulsion system; Reversible switchability.	^[^ ^31^ ^]^
	AOT, isooctane	Light	Microemulsion system; Rapid and fully reversible switchability.	^[^ ^33d^ ^]^
Pipeline	Amidines	CO_2_	Stabilize crude oil‐in‐water emulsions by cooperating with indigenous surfactants; CO_2_ triggers demulsification.	^[^ ^28^ ^]^
	RCOO^−^DMEAH^+^ (DMEA: *N*,*N*‐dimethylethanolamine)	CO_2_	Stabilize oil‐in‐water emulsions; Best performance at 65:35 oil/water volume ratio, 0.5 wt% DMEA concentration, 0.2 wt% NaCl concentration; Reversible CO_2_ switchability.	^[^ ^10c^ ^]^
	*N’*‐octyl‐*N,N*‐dimethylacetamidine/ *N’*‐dodecyl‐*N,N*‐dimethylacetamidine	CO_2_	Stabilize Xinjinag heavy oil‐in‐water emulsion; Emulsion is stable for heavy oil with a low asphaltene content and high acid number; Reversible CO_2_ switchability.	^[^ ^10c^ ^]^
Soil remediation	Sodium phenolate, Sodium carboxylate	CO_2_	Ex situ soil washing (Ottawa sand artificially contaminated with North Sea crude oil); Removal of oil contaminates from sand: 97%; Removal of oil contaminates from water: 95%; Residue surfactant on sand surface: 0.45%; Residue surfactant in water: 0.48%; Facile oil separation after CO_2_ bubbling.	^[^ ^39a^ ^]^
	DTMG	CO_2_	DTMG·CO_2_ shows a strong solubilization capacity for PAH; More than 50% PAH in surfactant solution could be released by bubbling N_2_ at 80 °C.	^[^ ^40^ ^]^
	DUSNa	CO_2_	Recovery of mineral: 92.1–94.1%; Residual oil on sands: < 0.17% (to the total mass); TOC, COD < 40 mg L^−1^.	^[^ ^41^ ^]^

### 2.3 Switchable Polymeric Surfactants

#### 2.3.1 Switchable Polymeric Surfactants for Oil Recovery

Polymeric surfactants are macromolecules with interfacial activity due to the presence of both hydrophilic and hydrophobic compositions.^[^ ^20^ ^]^ Compared to low‐molecular‐weight surfactants, polymeric surfactants feature strong modification capability in both chemical composition and molecular architecture. Moreover, polymeric surfactants are of particular interest for EOR due to their intrinsic property of increasing fluid viscosity, as well as their rheological behaviors. In fact, polymeric surfactants have been extensively used as processing aids in EOR, even without being recognized for decades.^[^ ^20^, ^46^ ^]^ In the following section, we focus on the switchable polymers aimed at providing functionalized interfacial activity. The responsive polymers designed for switching solution viscosity will be discussed in Section 3.2.

Thermo‐responsive polymeric surfactants with switchable interfacial activity have been investigated for the water‐based oil sands extraction process.^[^ ^7^, ^47^ ^]^ Thermo‐responsive polymers are known for exhibiting a dramatic change of water solubility when the environment temperature bypasses a critical point.^[^ ^48^ ^]^ If the polymer is insoluble at a lower temperature and becomes soluble at a higher temperature, the transition temperature is known as the lower critical soluble temperature (LCST). On the other hand, if the polymer becomes insoluble at a higher temperature, the transition temperature is referred to as the upper critical soluble temperature (UCST). Based on these phenomena, switchable polymeric surfactants could be fabricated by copolymerizing a block of thermo‐responsive polymer with another block of balancing polymer. For example, if the thermo‐responsive block has an LCST‐type behavior while the balance block is also water‐soluble, the synthesized block copolymer would be water‐soluble at a lower temperature. Once the temperature goes above the corresponding LCST point, the thermo‐responsive block switches to water‐insoluble while the balancing block remains unchanged, which collectively generates a transient amphiphilicity.

**Figure 4:**
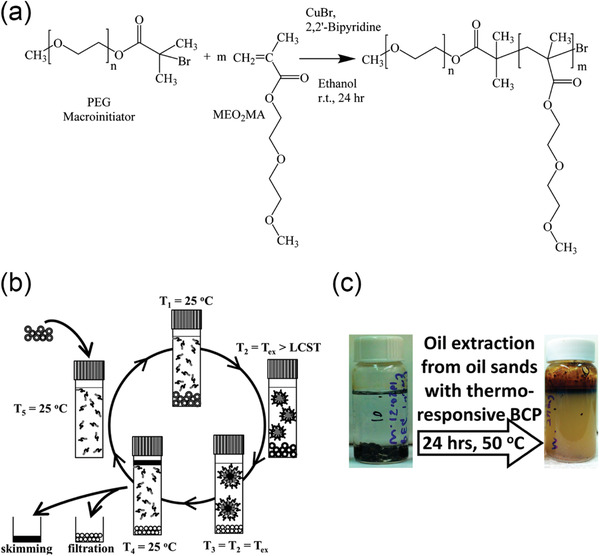
a) Polymerization of a thermal‐responsive polymeric constituted of a poly(ethylene glycol) (PEG) and a poly(2‐(2‐methoxyethoxy) ethyl methacrylate) (PMEO_2_MA) block surfactant (PEG‐*b*‐PMEO_2_MA). b) Proposed concept of thermal‐responsive polymeric surfactants enhancing bitumen recovery in oil sands extraction. The designed polymeric surfactants are anticipated to be completely water‐soluble at ambient temperature, while become interfacially active at the extraction temperature (*T*_ex_ > LCST) to improve the liberation of bitumen. After the extraction, when the environment temperature cools down, these polymeric surfactants would desorb from the oil‐water interface spontaneously and partition into the aqueous phase for the next cycle. c) Bottle tests of oil sands extraction using PEG‐*b*‐PMEO_2_MA. A significant amount of bitumen was extracted and floated to the top layer, leaving clean pristine sands at the bottom. a–c) Reproduced with permission.^[^ ^7d^ ^]^ Copyright 2015, American Chemical Society.

The concept of thermo‐responsive polymeric surfactants in a traditional water‐based oil sands extraction process is shown in **Figure** **4**. At a high temperature (*T* > LCST), polymeric surfactants are interfacially active and facilitate the liberation of ultraheavy oil (also known as bitumen) from sand grains. After a sufficient time of agitation to allow most of the bitumen to be released, the bulk temperature is cooled down to switch off the interfacial activity of polymeric surfactants. Eventually, bitumen products can be harvested by skimming, and clean solids are also filtered out at the bottom layer. It was confirmed that a significant enhancement of overall bitumen recovery could be achieved in small bottle tests^[^ ^7d^ ^]^ and at bench‐scale demonstrations.^[^ ^47a,c^ ^]^ Although these approaches are of great scientific importance, as well as opening new avenues for switchable polymeric materials in the oil recovery, the commercialization of thermo‐responsive polymeric surfactants is still challenged by the energy consumption to change the environment temperature, especially when considering the large scale of the oil sands industry. Besides, the costs of synthesizing responsive materials are not affordable for most operations since the current techniques of block copolymerization are still expensive.

Polymeric surfactants were also designed for chemical EOR application by introducing molecular recognition functions, which highlighted the switching behavior in the presence of oil molecules. Zou et al. synthesized a series of polyacrylamide (PAM) with different contents of *β*‐cyclodextrin (*β*‐CD) side groups.^[^ ^49^ ^]^ These polymers were injected into the petroleum stratum in the formation of the surfactant‐polymer complex with the surfactant molecules sitting inside the cavity of *β*‐CD. When coming into contact with the free crude oil, surfactant molecules would be replaced by the oil molecules due to hydrophobic interaction. Consequently, surfactants would be delivered and released specifically at the oil‐rich region, whereas the expelled oil molecules could be captured and carried by the hydrophobic cavity of *β*‐CD or by the PAM backbone. Such a “controlled” release of surfactants prevents the surfactant loss caused by solid adsorption, enhances the salinity tolerance, and reduces the dosage of conventional surfactants. Similarly, the same group extended their concept and further synthesized a novel hydrophobically associating acrylamide copolymer (HCMPAM) by introducing the *β*‐CD group in conjunction with the triblock copolymer, aimed at developing an efficient oil‐displacing agent adapted to the high‐temperature and high mineralization oil fields.^[^ ^50^ ^]^ It was demonstrated that HCMPAM could enhance 5.7–9.4% of oil recovery compared to the commercial partially hydrolyzed polyacrylamide (HPAM). Shakhvorostov et al. also synthesized a series of oil‐responsive betaines (also referred to as polyzwitterions or polymeric soap) that bear long alkyl chains as the prototype alkaline‐polymer‐surfactant (APS) flooding agent.^[^ ^51^ ^]^ The efficiency of the hydrophobic polymeric betaine that bears a C_16_H_33_ side group (poly(hexadecylaminocrotonate) betaine, PHDACB) was further investigated for the enhancement of heavy oil recovery in the Karazhanbas oilfield (Western Kazakhstan).^[^ ^51a^ ^]^ In a basic environment (e.g., KOH solution), PHDACB can exist in the form of micro‐/nano‐sized vesicles and micelles with a hydrophobic core and hydrophilic edges. The polymer could change its conformation in response to the presence of crude oil, where the long hydrophobic alkyl chain would be inverted and dipped into the oil droplets adsorbed on the rock, decreasing the oil‐water IFT and enhancing the sweeping efficiency. Results from sand pack flooding experiments showed that the injection of PHDACB (0.5%) solution incorporated with a 0.5% KOH aqueous solution increase the oil recovery by 38%, demonstrating the significant potential for EOR from a high viscous oil field. Indeed, these concepts of “oil‐responsive” polymeric surfactants require further investigation to verify their feasibility.

### 2.4 Switchable Interfacial‐Active Particles

Over the past decade, numerous studies have shown the potential of using particles in the oil industry, including in exploration, drilling, production, and refining. Particles were designed as the in situ sensing materials to estimate reservoir temperature/pressure,^[^ ^52^ ^]^ delineate geological maps including rock tortuosity/fracture/faults and the bypassed oil location,^[^ ^53^ ^]^ monitor real‐time oil reservoir evolution,^[^ ^54^ ^]^ and detect the waterfront of displacing fluid.^[^ ^55^ ^]^ Particles with amphiphilic moieties were also explored to stabilize emulsions (known as the Pickering emulsions)^[^ ^56^ ^]^ or soften rigid films caused by asphaltenes.^[^ ^57^ ^]^ In the downstream operations, particles have also been investigated to serve as catalysts^[^ ^58^ ^]^ or reduce the fouling effect.^[^ ^59^ ^]^ In the following sections, we will mainly focus on the particles that serve as “particulate” surfactants in oil recovery and oily waste cleanup applications. Their recent advances are summarized in **Table** **2**.

**Table 2:** Recent advances in switchable/magnetic‐responsive particles for oil recovery and oily waste cleanup applications

Application	Particle	Trigger	Performance	Ref.
Oil recovery	SiO_2_‐HMDS/HMDA‐SDBS (HMDS: Hexamethyl disilazane; HMDA:Hexamethylene diamine)	Oil	Reduce water injection pressure by 57.4% and 39.6% for two types of rock cores, respectively.	^[^ ^65^ ^]^
Dewattering	M‐EC	Magnetic	Water removal: 80%; Recycled for 10 times.	^[^ ^70b^ ^]^
	M‐CME‐EC	Magnetic	Water removal: >90%; Recycled more than 95% of the particle.	^[^ ^70c^ ^]^
	M‐Janus nanoparticle	Magnetic	Water removal: >92.76%; Recycled for more than 5 times.	^[^ ^70e^ ^]^
Oily wastewater treatment	SiO_2_ NPs in water, C*_n_* PMA in oil	CO_2_	In situ modification of SiO_2_ NPs to form stable Pickering emulsions; Reversible CO_2_ switchability; Surfactant concentration window is closely related to the length of hydrophobic tail.	^[^ ^75b^ ^]^
	SiO_2_ NPs and C_12_A in water (C_12_A: *N,N*‐dimethyl‐*N*‐dodecylamine)	CO_2_	In situ modification of SiO_2_ NPs to form stable Pickering emulsions; Reversible CO_2_ switchability;	^[^ ^75c^ ^]^
	Poly(acrylamide‐*co*‐acrylic) nanogel	pH	Stabilize crude oil‐in‐water Pickering emulsion; Demulsification by adding NaOH solution; Demulsification of light oils and crude oils emulsions are relatively slow.	^[^ ^84^ ^]^
	PNIPAm and Poly(N‐isopropyl‐ methacrylamide) (PNIPMAm) microgels	Thermal	In situ microgel‐Pickering emulsion system; Tunable directional interaction with temperature; Easy up‐scaling.	^[^ ^86^ ^]^
	Phenylboronic‐modified microgels	Sugar	Stabilize oil‐in‐water emulsion with the presence of sugar.	^[^ ^87^ ^]^
	CNC‐POEGMA/PMAA	pH and thermal	Stabilize oil‐in‐water emulsions at high pH and ambient temperature; Demulsification at low pH or high temperature.	^[^ ^88^ ^]^
	Fe_3_O_4_‐SiO_2_/PNIPAm	Thermal and magnetic	Stabilize oil‐in‐water emulsions; Magnetic Fe_3_O_4_ cores allows directional movement of oil droplets; Demulsification at high temperature (50 °C).	^[^ ^93^ ^]^
	Hybrid PNIPAm‐co‐HEMA‐co‐MAA microgel containing Fe_3_O_4_ NPs	Thermal and magnetic	Remote control of emulsion stability.	^[^ ^94^ ^]^
	Fe_3_O_4_‐SiO_2_‐PDMAEMA (PDMAEMA: Poly(2‐dimethylaminoethyl methacrylate))	pH and magnetic	Stabilize oil‐in‐water emulsions; Demulsification at low pH; Magnetic‐responsiveness for facile recycling.	^[^ ^8c^ ^]^

Particles with partial wetting properties (i.e., dual wettability) are able to adsorb at the oil‐water interface. One of the most important characters of Pickering emulsions is that the adsorption of interfacially active particles at the oil‐water interface decreases the total system energy remarkably. Binks showed that the energy difference (Δ*E*) to remove a particle from the interface is given by the following equation, if assuming that the particle size is small enough (<2 µm) to neglect their gravitational effects:^[^ ^60^ ^]^

(3) $$\Delta E=\pi r^{2}\gamma_{\mathrm{ow}}{\left(1\pm \cos\theta_{w}\right)}^{2}$$

where *r* is the radius of the particle, *γ*_ow_ is the interfacial tension of the pure water and pure oil, and *θ*_w_ is the particle‐oil‐water three‐phase contact angle measured in the water phase. The plus‐minus sign (±) represents the desorption of the particle from the interface into the oil phase (positive, “+”) or into the water phase (negative, “−”).^[^ ^60^, ^61^ ^]^ According to Equation (3), the desorption energy is calculated to be as high as 2750 *kT* for a particle of 10 nm and a contact angle of 90° at the toluene‐water interface, which is three orders of magnitude higher than that of a typical surfactant molecule.^[^ ^62^ ^]^ Thereby, interfacially active particles are mostly adsorbed at the interface firmly and irreversibly due to their relatively high energy penalty for desorption. This phenomenon is significantly different from the molecular surfactants that feature a dynamic equilibrium between adsorption and desorption at the interface.

More interestingly, interfacially active particles can be designed with certain switchability or magnetic‐responsivity in order to achieve a facile manipulation of their adsorption at the interface. For example, if the surface wettability of a particle (*r* = 10 nm) could be switched from a contact angle of 90° to 10°, the corresponding desorption energy (Δ*E*) would drop to as low as 0.63 *kT* for a particle partition into the water phase. Such a calculation indicates that the particle has a high tendency of leaving the interface and dispersing in the aqueous solution. Namely, the interfacial activity of the particle is switched off, and the corresponding emulsion would no longer be stable. The stimuli‐responsivenesss could be introduced either based on the surface characters of solid cores, e.g., graphene oxide response to bulk pH and irons oxide (Fe_3_O_4_) response to the magnetic field, or by introducing responsive functional groups, e.g., grafting poly(*N*‐isopropyl acrylamide) (PNIPAm) for thermal responsiveness. In a recent literature review, Tang et al. summarized the development of stimuli‐responsive particles and their potential application.^[^ ^21a^ ^]^ Obviously, there is a growing number of studies on stimuli‐responsive particles, and it is safe to say that the potential market in this field will increase rapidly in the next decade.

#### 2.4.1 Switchable Particles for Oil Recovery

Ideally, particles could be employed to EOR applications via the mechanisms of residual oil saturation reduction, interfacial modification, viscosity control, foam stabilization, wettability alternation, and conformation change.^[^ ^63^ ^]^ Although much efforts have been made to investigate the particle‐enhanced oil recovery techniques,^[^ ^64^ ^]^ there are only a few reports using the responsive particles. Liu et al. developed environment‐responsive silica (ERS) nanoparticles (NPs) capable of releasing functionalized silica particles using environmental variations, e.g., increasing the concentration of inorganic acids or salts, and thereby decreasing the hydrophobicity of pore surfaces near the oil‐rich region.^[^ ^65^ ^]^ Consequently, the resistance to water injection was dramatically reduced, and the overall oil recovery ratio was improved in the ultra‐low permeability reservoir. Soares et al. utilized 2‐D pore‐scale simulation confirming that the presence of magnetic‐responsive NPs in collaboration with an external magnetic field could help to overcome strong capillary pressure and displace oil ganglia.^[^ ^66^ ^]^ The improved oil displacement was attributed to the induced magnetic pressure arising with the magnetization.

**Figure 5:**
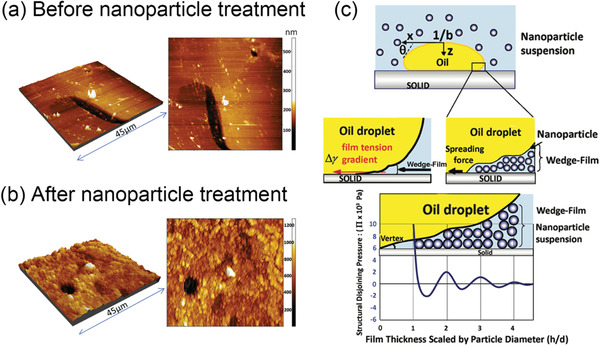
Topography picture of calcite surfaces a) before and b) after nanoparticle treatment. a,b) Adapted with permission.^[^ ^67d^ ^]^ Copyright 2015, Elsevier. c) Schematic illustrations of the wedge‐shaped nanoparticle structure and the structural disjoining pressure. Nanoparticles can form a ordered structure of wedge film near the three‐phase contact line, which promotes the spreading of the phase containing nanoparticles. The corresponding structural disjoining pressure is normal to the solid‐liquid interface and is higher near the vertex of the wedge film. Reproduced with permission.^[^ ^69^ ^]^ Copyright 2011, American Chemical Society.

To date, the biggest challenge of applying responsive particles for EOR applications is still the lack of fundamental understanding of their behaviors in complex reservoir conditions. In the EOR process, several mechanisms have been proposed for particles enhancing heavy oil liberation, including wettability alternation, IFT reduction, and disjointing pressure change.^[^ ^64^, ^67^ ^]^ Firstly, particles are proposed to be able to alter the rock wettability from hydrophobic to hydrophilic by adsorbing onto the solid surface (coating mechanism) and/or removing the original adsorbed molecules (cleaning mechanism).^[^ ^67b^ ^]^ Such a surface wettability alternation is most likely to switch the oil‐solid interaction from attractive to repulsive. Al‐Anssari et al. investigated calcite surfaces by atomic force microscopy (AFM) before and after the nanoparticle flooding treatment.^[^ ^67d^ ^]^ Experimental images clearly indicated that particles had a dramatic influence on the surface morphology (**Figure** **5**a,b). Secondly, it is also well‐known that the presence of particles could decrease the oil‐water IFT, which is essential to the liberation of heavy oil from solids.^[^ ^68^ ^]^ The relationship between oil‐water IFT and oil recovery has been discussed in Section 2.1. In general, lower oil‐water IFT values improves the oil detachment from hydrophilic solid substrates. Lastly, particles have a tendency to spread at the oil‐water‐solid three‐phase contact line and form a wedge‐film when the oil‐solid interaction is repulsive, which increases the local disjoining pressure.^[^ ^67^, ^69^ ^]^ As illustrated in Figure 5c, the presence of particles could build up disjoining pressure up to 5 × 10^4^ Pa in the vertex region, facilitating the oil‐solid separation and allowing the fluid to spread even further.^[^ ^64^, ^67^ ^]^ Collectively, the wettability alteration mechanism is of most importance since it tunes the oil‐solid interaction into repulsive. Moreover, the IFT reduction and wedge‐film mechanisms further accelerate the oil‐solid separation process, as long as the interaction forces remain negative.

#### 2.4.2 Magnetic‐Responsive Particles for Demulsification

Magnetic‐responsive particles were also designed to remove the water droplets trapped in an oil product, especially after recovery processes involving water flooding/washing.^[^ ^70^ ^]^ Although the water content is often low (< 5%) in the oil products,^[^ ^22^ ^]^ a trace amount of water that contains salt could become detrimental to downstream operators, causing equipment corrosions and poisoning refinery catalysts.^[^ ^71^ ^]^ Hence, it is mandatory to eliminate the dispersed water droplets in the form of water‐in‐oil (W/O) emulsions before sending them to upgraders. Magnetic‐responsive particles are widely considered to be the most promising candidates to remove residual water in a facile manner. Strictly speaking, magnetic‐responsive interfacial‐active particles do not switch off their surface activity with an external magnetic field. Instead, they adsorb onto the oil‐water interface and drive the tagged water droplets moving in the direction of the externally applied magnetic field. Consequently, the unwanted droplets (e.g., the water content in the production oil) could be concentrated near the magnet, whereas the particles could then be recycled easily. The breakdown of emulsions stabilized by magnetic‐responsive particles could be achieved eventually when the strength of the magnetic field is high enough to overcome the desorption energy of the particles at the interface.^[^ ^72^ ^]^

**Figure 6:**
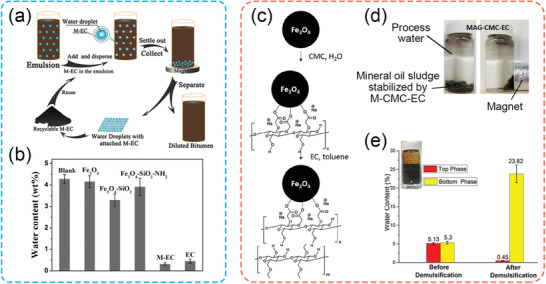
a) Process for the demulsification and recycling tests using M‐EC as demulsifiers; and b) Dewatering efficiency from water‐in‐diluted bitumen emulsions with various types of magnetic NPs. a,b) Reproduced with permission.^[^ ^70b^ ^]^ Copyright 2011, American Chemical Society. c) Preparation routine for M‐CMC‐EC NPs using sequential adsorption method; d) Magnetic‐responsive behavior of the mineral oil sludge stabilized by M‐CMC‐EC NPs; and e) Water content of emulsions before and after demulsification using MAG‐CMC‐EC nanoparticles. c–e) Adapted with permission.^[^ ^70c^ ^]^ Copyright 2018, American Chemical Society.

Various magnetic‐responsive particles have been developed to remove residual water from diluted bitumen in the froth treatment process in the Canadian oil sands industry.^[^ ^70a–c,e^ ^]^ The main objective was to exploit the magnetic‐responsive and interfacially active particles replacing the rigid asphaltene film that covers the water droplets, thereby tagging the undesired water droplets for magnetic separation. The strategies involved the coating of functional materials (e.g., ethylcellulose (EC), or polyelectrolytes on the magnetic‐responsive core (e.g., Fe_3_O_4_ NPs).^[^ ^73^ ^]^ Peng et al. fabricated the magnetic‐responsive particles by grafting poly‐EC on an iron core (Fe_3_O_4_) throughout the esterification reaction (denoted as M‐EC, where “M” refers to the magnetic‐responsive core).^[^ ^70a,b^ ^]^ M‐EC particles did an excellent job on disrupting the aged asphaltene films and removed more than 80% of the water (**Figure** **6**a,b). Also, these novel M‐EC particles could be recycled ten times without a noticeable decrease in the dewatering efficiency. Following their pioneering works, Liang et al. further developed a facile routine to prepare EC‐coated iron particles. Bare iron oxide (Fe_3_O_4_) NPs were first primed with sodium carboxymethyl cellulose (CMC), followed by a coating of ethyl cellulose (EC) on the CMC‐primed nanoparticles (M‐CMC‐EC), both through a physical adsorption process in different solutions (Figure 6c–e).^[^ ^70c^ ^]^ CMC was utilized as the bridging materials due to their affinity to both the mineral surface and EC.^[^ ^74^ ^]^

#### 2.4.3 Switchable/Magnetic‐Responsive Particles for Oily Waste Cleanup

Interfacial‐active switchable particles are also emerging materials for oily wastewater treatment. Compared to switchable surfactants, using switchable particles for oily wastewater treatment process are considered to be more environmentally benign. In general, switchable particles could be applied to adsorb onto the oil‐water interface, substitute the natural/original surfactants, and form stable Pickering emulsions. Once the switchable particles fully dominate the interfacial properties of the oil‐water interfaces, a facile separation of the oily pollutants from the water body could be achieved with ease by switching off the interfacial activity of the switchable particles. One of the most studied routine for synthesizing switchable particles is by grafting switchable functional groups on the surface of a rigid core particle. There exists a wide selection of the base particles, including SiO_2_,^[^ ^60^, ^72^, ^75^ ^]^ metal oxide,^[^ ^76^ ^]^ clay,^[^ ^77^ ^]^ and carbon,^[^ ^78^ ^]^ as well as various methodologies to endow particles with switchable functionality.^[^ ^21^, ^79^ ^]^

**Figure 7:**
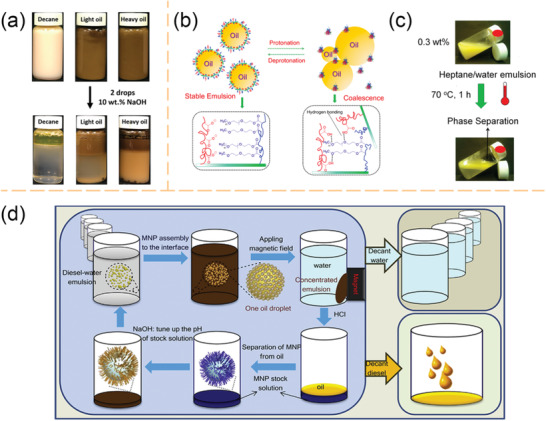
a) Demulsification of O/W Pickering emulsions stabilized by pH‐responsive nanogels by adding two drops of 10 wt% NaOH solution. a) Reproduced with permission.^[^ ^84^ ^]^ Copyright 2019, Elsevier. b) Proposed mechanism of the pH‐responsive behavior of the Pickering emulsions stabilized by cellulose nanocrystals grafted with poly(oligoethylene glycol) methacrylate and poly(methacrylic acid) (CNC‐POEGMA‐PMAA). c) Heptane‐in‐water emulsions stabilized by CNC‐POEGMA‐MAA (0.3 wt%) and its thermo‐responsive behavior when being placed in a 70 °C water bath for 1 h. b,c) Adapted with permission.^[^ ^88^ ^]^ Copyright 2016, American Chemical Society. d) Application of pH‐responsive magnetic nanoparticles (MNPs) as recyclable stabilizers for oil‐water separation. Reproduced with permission^[^ ^8c^ ^]^. Copyright 2015, Elsevier.

Microgels formed by crosslinked polymer networks have been investigated as a new type of particulate emulsifier to stabilize O/W Pickering emulsions.^[^ ^17^, ^80^ ^]^ Compared to the rigid particles, it has been demonstrated that soft particles like microgels are more interfacially active, attributed to their deformability at the oil‐water interface.^[^ ^81^ ^]^ In fact, the microgels behave more similarly to polymeric surfactants during the emulsification process.^[^ ^80b^ ^]^ More specifically, microgels made of stimuli‐responsive polymers could exhibit switchable interfacial activity, thus allowing the remote control of the emulsion stability. Fabrication of dual‐responsive microgels was reported by copolymerizing NIPAm and methacrylic acid (MAA) monomers^[^ ^82^ ^]^ and in‐depth revealed their adsorption mechanisms at the oil‐water interface.^[^ ^7^, ^83^ ^]^ Such Pickering emulsions could become unstable when the environment temperature raised above the LCST of PNIPAm or when the aqueous solution was acidified. Recently, Geng et al. reported the crude oil‐in‐water Pickering emulsions stabilized by pH‐responsive nanogel comprising nano‐sized crosslinked poly(acrylamide‐co‐acrylic). The demulsification of these Pickering emulsions could be triggered by adding two drops of 10 wt% NaOH solution.^[^ ^84^ ^]^ The decane/water Pickering emulsion achieved complete phase separation in 48 h, whereas the creaming of both light oils and crude oils is relatively slow (**Figure** **7**a). One of the explanations of this undesired phenomenon is that the basic environment would cause the ionization of asphaltenes in the crude oil to form amphoteric surfactants.^[^ ^85^ ^]^ In addition, Månsson et al. developed an in situ routine to assemble PNIPAm‐based microgels into colloidal molecules.^[^ ^86^ ^]^ The thermo‐responsiveness introduced by PNIPAm sites permits facile control of the interaction between assembled colloidal molecules from soft repulsive to short‐range attractive. Besides, Tatry et al. also investigated phenylboronic‐modified microgels that could stabilize O/W emulsions in the presence of saccharide, i.e., sugar‐responsive microgels.^[^ ^87^ ^]^

Biomass materials, such as cellulose nanocrystals (CNCs) derived from the acid hydrolysis, have recently received considerable attentions as biocompatible particle cores for the wastewater treatment due to their sustainable and eco‐friendly features. Tang et al. investigated dual‐responsive functionalized CNCs for surfactant‐free oil harvest.^[^ ^88^ ^]^ Poly(oligoethylene glycol) methacrylate (POEGMA) and poly(methacrylic acid) (PMAA) were grafted onto CNC‐NPs to endow thermo‐ and pH‐responsive characters, respectively. These modified CNC‐NPs diffuse onto the oil‐water interface and stabilize emulsions at high pH and ambient temperatures. Meantime, a robust separation of oil from the aqueous phase was achieved by either decreasing the bulk pH or raising the environmental temperature (Figure 7b,c). The authors hypothesized that the chain flexibility and molecular weight of PMAA play important roles in the destabilization of the emulsions.^[^ ^88^ ^]^ Since CNCs originate from biological products, and the modified NPs can be recycled for at least five times without any loss in efficacy. This dual‐responsive CNC NP is considered to be sustainable nanomaterials for crude oil transportation and oily wastewater treatments. Besides, there are various reports on the responsive particles using other common bio‐materials, such as chitosan^[^ ^89^ ^]^ and starch.^[^ ^90^ ^]^

Another direction of developing particles for oily wastewater treatment is the use of magnetic‐responsive and interfacially active particles. These materials are increasingly popular of easy to recycle. There are two common strategies for fabricating magnetic‐responsive particles in wastewater treatments: grafting a biocompatible outer layer with specific functions onto a magnetic‐responsive core,^[^ ^8^, ^91^ ^]^ or grafting magnetic‐responsive NPs onto a biodegradable carrier.^[^ ^92^ ^]^ Although the preparation routine differs, these magnetic‐responsive particles are both designed to collect dispersed oil droplets in the aqueous environment and to concentrate the oily pollutants by using a magnetic field. For example, Chen et al. studied polymer‐grafted magnetic composite particles for oil‐water separation and oil droplet transportation.^[^ ^93^ ^]^ In an effort to harvest the oil components after the magnetic collection, a secondary switchable group is commonly involved to breakdown the Pickering emulsions and recycle both oil and particles. Brugger and Richtering fabricated magnetic‐/thermo‐sensitive microgels for the remote control of separation and stability of the O/W Pickering emulsions.^[^ ^94^ ^]^ Wang and co‐workers also reported pH‐responsive magnetic nanoparticles (MNPs) for efficient emulsification of crude oil‐in‐water and facile oil‐water separation (Figure 7d).^[^ ^8c^ ^]^

#### 2.4.4 Switchable/Magnetic‐Responsive Janus Particles in Applications

Janus particles are a specific type of colloidal particles that feature asymmetric surface property, with one side being more hydrophilic and the other side being more hydrophobic. Janus particles with such a heterogeneous surface wettability could adsorb at the oil‐water interface even stronger than homogeneous interfacial‐active particles, and thereby lead to the formation of ultra‐stable emulsions.

**Figure 8:**
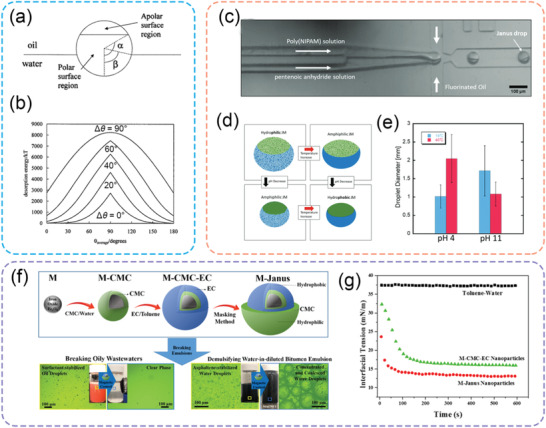
a) Schematic illustration of a Janus particle at the oil‐water interface. b) Variation of particle desorption energy with are a weighted average contact angle for particles of radius 10 nm and *α* = 90° and the oil‐water tension of 36 mN m^−1^. a,b) Adapted with permission.^[^ ^95a^ ^]^ Copyright 2001, American Chemical Society. c) Janus droplet formation of polymer precursor solutions via shear by fluorinated oil in microfluidic flow‐focusing device. d) Switching behavior of the dual‐responsive Janus particle. e) Droplet diameter of the emulsion stabilized by dual‐responsive Janus particle as a function of environmental pH and temperature. c–e) Adapted with permission.^[^ ^100^ ^]^ Copyright 2020, The Royal Society of Chemistry. f) Top: Synthesis procedures of M‐Janus NP; Bottom: Demulsification of oily wastewater and water‐in‐diluted bitumen emulsions; g) Dynamic IFT of the toluene‐water interface with homogeneous M‐CMC‐EC NPs and M‐Janus NPs. f–g) Reproduced with permission.^[^ ^70e^ ^]^ Copyright 2019, Elsevier.

**Figure** **8**a is a schematic illustration of a Janus particle locating at the planar oil‐water interface. In order to obtain a comprehensive understanding of the adsorption of Janus particles, the relative areas of the polar and apolar regions in a Janus particle are parameterized by the angle *α*, whereas the immersion depth of the particle in the oil‐water interface is characterized by the angle *β*. Correspondingly, the total free energy for removing a Janus particle from the interface can be described by the following equations^[^ ^95^ ^]^

For *β* ≤ *α*:

(4) ΔEβ=2πr2γao1+cosα+γpocosβ−cosα12+γpw1−cosβ−12γowsin2β

For *β* ≥ *α*:

(5) ΔEβ=2πr2γao1+cosβ+γawcosα−cosβ12+γpw1−cosα−12γowsin2β

where *r* is the radius of the Janus particle, where *γ*_ao_, *γ*_po_, *γ*_pw_, *γ*_aw_, and *γ*_ow_ are the interfacial energies of the apolar‐oil, polar‐oil, polar‐water, apolar‐water, and oil‐water interfaces, respectively. Accordingly, the contact angles of both the apolar side (*θ*_a_) and polar side (*θ*_p_) of the Janus particle can be given by Young's equation^[^ ^96^ ^]^

(6) $$\cos\theta_{a}=\left(\gamma_{\mathrm{aw}}-\gamma_{\mathrm{ao}}\right)/\gamma_{\mathrm{ow}}$$

(7) $$\cos\theta_{p}=\left(\gamma_{\mathrm{pw}}-\gamma_{\mathrm{po}}\right)/\gamma_{\mathrm{ow}}$$

Subsequently, the desorption energy of a Janus particle can be calculated and compared by its average contact angle (*θ*_average_),^[^ ^95a,b^ ^]^ which is defined as the following

(8) $$\theta_{\mathrm{average}}=\frac{1}{2}\left(\theta_{a}\left(1+\cos\alpha \right)+\theta_{p}\left(1-\cos\alpha \right)\right)$$

Figure 8b shows the calculation results of the desorption energy of a Janus particle with *α* = 90°, *r* = 10 nm, and *γ*_ow_ = 36 mN m^−1^.^[^ ^95a,b^ ^]^ The wettability difference between the apolar and polar faces of the Janus particle is demonstrated by the value of Δ*θ*, which is defined as Δ*θ* = (*θ*_p_ – *θ*_a_)/2. When Δ*θ* = 0°, the particle can be considered as a homogeneous particle since there is no wettability difference between the two faces. The result demonstrated that Janus particles (with Δ*θ* > 0°) always perform better interfacial activity than homogeneous particles from the perspective of desorption energy. On the other hand, the largest value of desorption energy is obtained from the Janus particle with the highest possible Δ*θ* value (90°), which means that one side of the particle is superhydrophilic (*θ*_a_ = 0°) and the other side is superhydrophobic (*θ*_a_ = 180°). Under such a circumstance, the desorption energy is about three‐fold higher compared to the homogeneous particle, illustrating the superb interfacial activity of such Janus particles.

##### Switchable Janus Particles

Janus particles with switchable interfacial activity have been considered with great potentials from both scientific and industrial perspectives. The key to fabricating switchable Janus particles relies on disrupting or diminishing the wettability differences between the two faces of the Janus particles, i.e., reduce the value of Δ*θ*. This can be achieved by implementing switchable functional groups on one side of the Janus particles. As illustrated by Figure 8b, decreasing Δ*θ* leads to the reduction of desorption energy. Consequently, Janus particles are much easier to desorb from the interface, and its corresponding emulsions become less stable. Indeed, there are already various approaches being documented to fabricate robust Janus particles that could respond to various types of external stimuli, such as pH^[^ ^97^ ^]^ and light.^[^ ^98^ ^]^

More recently, Janus particles with multiple switching triggers have also been reported with more complicated phase behaviors. Chen et al. reported a facile routine of obtaining Janus nanoplates by crushing the hollow spheres assembled by thermo‐/pH‐responsive block copolymers.^[^ ^99^ ^]^ The microfluidic devices were used to fabricate the Janus microgels comprised of the separated network between thermo‐responsive PNIPAm and pH‐responsive poly(anhydride) (Figure 8c–e).^[^ ^100^ ^]^ These dual‐responsive Janus particulate emulsifiers could be tuned into the amphiphilic state at low pH low temperature or at high pH high temperature. In all other scenarios (low pH high temperature, or high pH low temperature condition), their desorption energy is weakened due to the disruption of hydrophilic‐hydrophobic balance in their Janus structure, causing the increase of droplets diameter and eventually demulsification. To the best of our knowledge, however, the difficulties in fabrication of Janus particles are still the biggest challenge for further investigation in large‐scale applications.

##### Magnetic‐Responsive Janus Particles

Janus particles also possess promising synergistic interaction if cooperating with magnetic responsivity. Since Janus particles have much higher desorption energy than homogeneous particles, it is possible that Janus particles are less likely to desorb from the interface under a moderate level of the magnetic field. Therefore, emulsions stabilized by magnetic‐responsive Janus particles have better stability than magnetic‐responsive homogeneous particles. These emulsions could be transported or collected by the external magnetic field, with a minimized probability of droplets coalescence.

He et al. developed the interfacial activity of magnetic‐responsive particles by synthesizing magnetic‐responsive Janus particles (denoted as M‐Janus).^[^ ^70e^ ^]^ Two natural cellulose materials with opposite solubilities (hydrophobic EC and hydrophilic CMC) were coated heterogeneously on the magnetite NPs surface (Figure 8f,g). These M‐Janus NPs exhibited a much stronger interfacial activity and could firmly anchor at the toluene‐water interface, preventing the coalescence of water droplets during the transfer and the movement caused by the external magnetic field. M‐Janus NPs were also able to be recycled at least five times with phase separation efficiency higher than 95%. The same group of authors also applied these M‐Janus particles to remove waste‐oils from different wastewaters, that were originally stabilized by natural and/or synthetic surfactants.^[^ ^91^, ^101^ ^]^ These M‐Janus particles exhibited robust interfacial activity and magnetic response in high salinity conditions, such as tap water and seawater. Therefore, the authors concluded that their novel M‐Janus particles were supposed to have potential in a wide range of applications directly without further customization. Besides, Kim and co‐workers presented a straightforward and robust routine for the preparation of magnetic‐patchy Janus colloidal particles, where seeded monomer swelling and polymerization were employed in a batch reaction.^[^ ^102^ ^]^ Song et al. also successfully fabricated claw‐shaped M‐Janus particles with a convex hydrophilic surface and a concave hydrophobic surface by emulsion interfacial polymerization followed by selective electrostatic assembly.^[^ ^103^ ^]^ The authors demonstrated that the unique shape of such M‐Janus particles has a pronounced effect on promoting the coalescence of oil droplets. Microscale oil droplets were separated from water within 120 s with a separation efficiency >99%.

## 3 Switchable Viscosity

### 3.1 Role of Viscosity

**Scheme 2:**
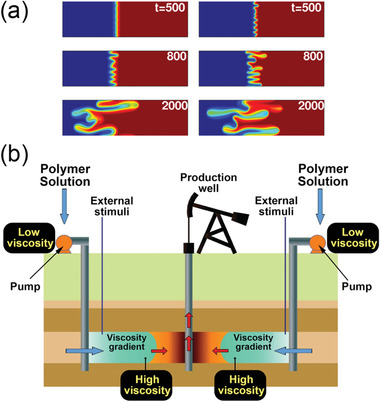
a) Simulated viscous fingering effect between two immiscible liquids. Reproduced with permission.^[^ ^104b^ ^]^ Copyright 2017, AIP Publishing. b) Illustration of using the stimuli‐responsive polymer with switchable viscosity as the displacing fluid in EOR. Adapted with permission.^[^ ^106^ ^]^ Copyright 2014, Society of Petroleum Engineers.

When displacing the original crude oil by in situ waterflooding, it is common seen for water to flow much faster than the oil phase due to viscosity differences, leaving most of the oil products behind without being recovered. This phenomenon is known as the viscous fingering (**Scheme** **2**a). It dramatically influences the in situ flow behavior and typically negatively impacts on the recovery ratio.^[^ ^2^, ^104^ ^]^ Mobility ratio (*M*) is widely used in the literature to describe the displacing efficiency and for the preliminary screening of the viscous fingering effect. Mobility ratio is defined by the following equation based on Darcy's Law:^[^ ^105^ ^]^

(9) $$M\;=\frac{\lambda_{w}}{\lambda_{o}}\;=\frac{K_{\mathrm{rw}}}{\mu_{w}}/\frac{K_{\mathrm{ro}}}{\mu_{o}}\;$$

where *λ*_w_ and *λ*_o_ are the mobility of the water phase and oil phase, respectively; *K*_rw_ and *K*_ro_ are the relative permeability of the water phase and oil phase (mD), respectively; *μ*_w_ and *μ*_o_ are the viscosity of the water phase and oil phase (Pa s), respectively. The viscous fingering effect is less likely to occur when the mobility ratio is less than unity (*M* ≤ 1), which could be achieved by increasing the water viscosity (*μ*_w_) by adding viscosifiers (e.g., polymers).^[^ ^2^, ^106^ ^]^

It should be noted that Equation (9) is only validated for the reservoir without a previous water breakthrough. In reality, however, polymer‐assisted flooding (polymer‐EOR) is often initiated after water flooding. Under these circumstances, the mobility ratio (*M*_po_) during polymer‐EOR should be rewritten as the following equation^[^ ^2c^ ^]^

(10) $$M_{\mathrm{po}}=\frac{\lambda_{\mathrm{rp}}}{\lambda_{T}}\;=\frac{K_{\mathrm{rp}}}{\mu_{p}}/\left(\frac{K_{\mathrm{ro}}}{\mu_{o}}+\frac{K_{\mathrm{rw}}}{\mu_{w}}\right)$$

where *M*_po_ is the relative mobility ratio of the polymer solution; *λ*_rp_ is the relative mobility of the polymer solution; *λ*_T_ is the total relative mobility of the oil/water bank; *K*_rp_ is the relative permeability of the polymer solution; and *μ*_p_ is the viscosity of the polymer solution. Nevertheless, Equation (10) shows that increasing the viscosity of the polymer solution (*μ*_p_) is still imperative to reduce the relative mobility ratio (*M*_po_).

Polymer‐EOR has been reported to perform well in the reservoirs with oil viscosity between 10 to 150 mPa s.^[^ ^106^, ^107^ ^]^ However, the upper limit of oil viscosity is subjected to the injection difficulties, in which tremendous efforts is needed to pump in and out these highly viscous liquids. On the other hand, displacing fluids containing responsive viscosifiers could be designed with switchable viscosity that makes for an easier flow during the pumping stages while also capable of explicitly depositing high viscosity in the oil‐rich regions. The concept is schematically illustrated in Scheme 2b. External stimuli are introduced in situ to trigger the increase of fluid viscosity, such that the fingering effect is diminished, and the oil recovery is enhanced.

### 3.2 Switchable Polymeric Viscosifier

#### 3.2.1 Switchable Polymeric Viscosifier for Polymer‐EOR

Polymers are widely used to increase the viscosity of the displacing fluid in EOR applications. For example, PAM and its derivatives have been extensively studied for decades as effective viscosifiers to improve oil recovery from tertiary reservoirs.^[^ ^108^ ^]^ As discussed in Section 3.1, polymer solutions should have a comparable viscosity with the oil‐in‐place, which minimizes the viscous fingering effect and drives more crude oil toward the production well. However, as the oil‐in‐place becomes heavier in the late‐stage operation, it becomes extremely difficult and economically inefficient to inject the ultra‐viscous polymer solution. Moreover, polymer solutions usually exhibit a thermal‐thinning property and salt‐screening effect. In short, their viscosity is reduced at high temperature and high salinity, both of which, unfortunately, are also the in situ conditions in a petroleum reservoir. Therefore, the effectiveness of traditional polymer solutions becomes even weaker underground, limiting the extraction of crude oil with ultrahigh viscosity.

Switchable polymeric viscosifiers can stay at the “inactive” state (low viscosity) during the injection stage for better pumping efficiency while switching to the “active” state (high viscosity) when getting close to the oil bank in the reservoir. Therefore, successful implementation of responsive polymeric viscosifiers has the potential to better recover ultra‐heavy oil with fewer injection issues. Depending on the reservoir conditions and the process design, switching characters can be activated by the internal change (e.g., temperature elevation from the ground to the reservoir) or by external stimuli (e.g., secondary addition of acidic solutions).

**Figure 9:**
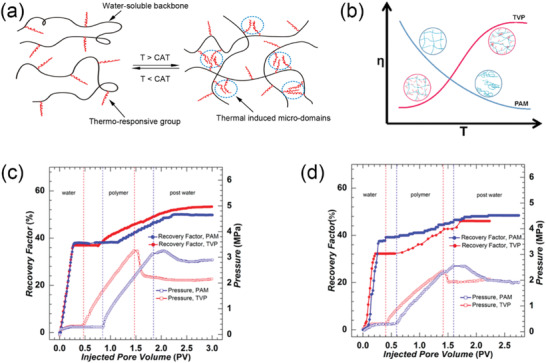
a) Mechanisms of thermoviscosifying behavior. Adapted with permission.^[^ ^110b^ ^]^ Copyright 1997, American Chemical Society. b) Illustration of the thermal‐induced viscosity change for PAM and TVPs. Recovery factors and flooding pressures using PAM or TVPs in core flooding tests at c) 45 °C and d) 85 °C (*C*_polymer_ = 0.2 wt%; Total dissolved solids = 101 000 mg L^−1^; Injected rate = 2 mL min^−1^). b–d) Reproduced with permission.^[^ ^111c^ ^]^ Copyright 2017, American Chemical Society.

During the past decade, thermoviscosifying polymers (TVPs) have been extensively investigated as the responsive polymeric viscosifiers to enhance oil recovery from hot reservoirs.^[^ ^11^, ^109^ ^]^ Unlike traditional polymer solutions, TVPs solutions exhibit an increased viscosity when the temperature rises above their critical associating temperature (CAT). Hourdet and his co‐workers first recognized the thermoviscosifying behaviors when grafting side chains with LCST behavior onto a water‐soluble polymer backbone.^[^ ^7^, ^110^ ^]^ Micro‐domains could be generated at high temperatures and interact with each other through hydrophobic interaction, which increased the solution viscosity due to entanglement (**Figure** **9**a,b). Such a counter‐intuitive thermal‐thickening behavior corresponds well with the demands in polymer‐EOR, where the polymer solutions can be less viscous in the pump‐in and pump‐out stages while switching to more viscous near the heavy oil reservoir. In addition, there is no need for external stimuli since the temperature gradient is readily embedded in the geological condition. Such a strategy of utilizing the internal stimuli is often referred to as “self‐adaptive” in the literature. Before reviewing the recent advances in TVPs, it is worth noting that the switching behavior of both TVPs and thermo‐responsive polymeric surfactants (as discussed in Section 2.3.1) relies on the LCST behaviors of thermo‐responsive polymers. However, they have different scopes of the switchable property, which differentiates their designing strategy and fields of applications. Thermo‐responsive polymeric surfactants, featuring switchable interfacial activity, are mostly applied to modulate oil‐water interfacial tension and control the emulsion stability. Meanwhile, the key character of TVPs is that they exhibit much higher solution viscosity at high temperatures, such that TVPs have the potential to reduce the viscous fingering effect in EOR applications. Furthermore, switchable polymeric surfactants are typical of a low degree of polymerization, whereas TVPs are often large macromolecules with a highly entangled network at high temperatures.

Feng's group developed a series of TVPs for EOR applications, from laboratory demonstrations to pilot‐scale implementations.^[^ ^111^ ^]^ Similar concepts were also conducted by other groups using various types of TVPs and under different reservoir conditions.^[^ ^112^ ^]^ Li et al. proved that TVPs exhibited better sweep efficiencies and mobility controls in the core flooding tests when compared with the conventional PAM addition (Figure 9c,d). More specifically, TVP solutions resulted in a higher increment in the oil recovery factor after water flooding (16.4% at 45 °C and 15.5% at 85 °C), whereas the increment of recovery factor by PAM solutions decreased at elevated temperatures (12.0% at 45 °C and 9.20% at 85 °C).^[^ ^111c^ ^]^ Interestingly, the thermoviscosifying behavior is less affected by the salt concentration compared to conventional polymers, suggesting better salinity tolerance of TVPs in an EOR operation.^[^ ^113^ ^]^

Although the potential of TVPs in polymer‐EOR has been demonstrated in various studies, there are also debates on their feasibilities in complex reservoir conditions and large‐scale operations. In order to perform thermoviscosifying behavior, TVPs have to reach a critical aggregation concentration (CAC) in the aqueous phase, below which the solution is thermo‐thinning.^[^ ^114^ ^]^ This is because the intermolecular interactions of TVPs chains are too weak to form an entangled network when the polymer concentration is lower than CAC values.^[^ ^114b^ ^]^ Instead, polymers tend to coil up into clusters due to intramolecular association between their side chains, reducing the hydrodynamic volume and thereby decreasing solution viscosity. Besides, the apparent CAC value of TVPs in a porous medium is also susceptible.^[^ ^109^, ^115^ ^]^ Even if the TVPs concentration exceeds their corresponding CAC value in the solution (e.g., in a semi‐diluted solution), their thermoviscosifying behavior is only validated within a certain temperature range.^[^ ^115^, ^116^ ^]^ When the temperature goes above a critical point, the entropic penalty of forming such organized network starts to influence the system, leading to a weaker strength of the super‐aggregate structures and a more vigorous motion of surrounding water molecules.^[^ ^11a^ ^]^ As a result, TVPs have a maximum viscosity at the critical temperature point and then exhibit thermal‐thinning behaviors in the regime above. Apart from those concerns, the cost of synthesizing branched copolymers still struggles against the economic outcomes for most operations at the current stage.^[^ ^111c^ ^]^

pH‐responsive polymers can also alter their solution viscosity by changing the solution pH. It is well‐known that polyelectrolytes with more than one charged group can change their conformation at different pH conditions, thereby affecting the solution viscosity. Typically, polyanions experience high viscosity at high pH and low viscosity at low pH, whereas polycations exhibit the opposite.^[^ ^11^, ^46^ ^]^ Tam and co‐workers designed pH‐responsive polyelectrolytes with a comb‐like structure, comprised of a random copolymer of PMAA, ethyl acrylate (EA), and hydrophobically modified macromonomers.^[^ ^8^, ^117^ ^]^ These polymer latexes exhibit swelling behavior at high pH conditions and significantly increase the solution viscosity. Araujo et al. demonstrated that the exceptional thickening capability of comb‐like polyelectrolytes is a combination of electrostatic repulsion, intra‐/inter‐molecular association, and EA block association.^[^ ^118^ ^]^ However, to the best of our knowledge, pH‐responsive polymers as a responsive viscosifier were not explored as extensively as the TVPs in EOR applications due to several technical difficulties. One problem was that the presence of dissolved ions would greatly reduce the viscosity of polyelectrolyte solutions due to the screening of charges.^[^ ^8a^ ^]^ The local mechanical stress also affected the formation of the polymer network, diminishing the polymer performance when bypassing the porous medium in the reservoir.^[^ ^119^ ^]^ In addition, the interaction between charged polyelectrolytes and natural surfactants have a pronounced influence on their thickening effect.^[^ ^46^, ^120^ ^]^

Several attempts have been made to develop switchable polymeric viscosifiers for EOR applications other than thermal or pH responses. Philippova and Khokhlov reported a “water‐responsive” polymer that could find the water influx by itself and block the unwanted flows of displacing fluids.^[^ ^121^ ^]^ Zhong et al. proposed a novel associative polymer with a good salt‐thickening effect, which also exhibited heat‐thickening and shear thickening properties at low shear stress.^[^ ^122^ ^]^ Zhang and co‐workers developed self‐adaptive polymers that respond to the applied stress.^[^ ^123^ ^]^ When flowing through the converging sections of the porous medium, polymer aggregations disassembled due to the local shear and elongated force, helping the displacing fluid enter the pore throat regions. In the diverging area, polymers regained intermolecular interactions and reinforced the solution viscosity. Further investigations are needed to assess the viability and feasibility of these novel switchable polymers.

Despite the specific issues described in the previous paragraphs, each type of switchable polymeric viscosifier also suffers from some generic concerns when polymers are used in EOR applications.^[^ ^2^, ^124^ ^]^ Polymer retention is one of the major challenges in polymer‐EOR processes, which is consisted of three main mechanisms: polymer adsorption, mechanical entrapment, and hydrodynamic retention.^[^ ^108^, ^125^ ^]^ Since polymers are large molecules and are usually rich in functional groups, they can bind with solid surfaces via reversible physical adsorption and/or irreversible chemisorption, be mechanically retained in narrow pore throats, or hydrodynamically trapped in stagnant zones. Collectively, a higher dosage of polymers is required to compensate for the loss of viscosifiers before the oil bank,^[^ ^126^ ^]^ whereas a considerable amount of oil‐in‐place might be blocked in the low permeability zones due to the plug of pore throats. In general, polymer retention has a tremendous impact on overall oil recovery ratio and economic feasibility. A comprehensive understanding of the polymer retention mechanisms was recently reviewed by Al‐Hajri et al.^[^ ^2c^ ^]^ Nevertheless, there is still a lack of studies in the interdisciplinary area of switchable polymers and their retention in porous media at different states of viscosity. It is of both scientific and practical importance to obtain fundamental knowledge when designing switchable polymeric viscosifiers for EOR applications.

### 3.3 Switchable Self‐Assembled Viscosifier

#### 3.3.1 Switchable Self‐Assembled Viscosifier for EOR

**Figure 10:**
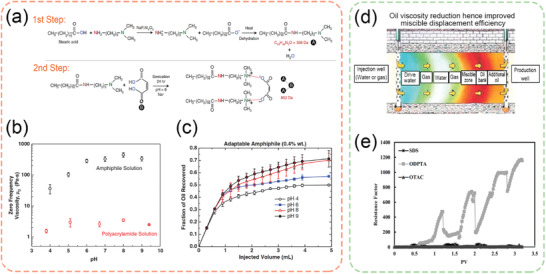
a) Two‐step synthesis protocol for the preparation of pH‐responsive amphiphiles. b) Effect of solution pH on the viscosity of amphiphile solution. c) Fraction of oil recovered using the amphiphile at different pH values. a–c) Reproduced with permission.^[^ ^106^ ^]^ Copyright 2014, Society of Petroleum Engineers. d) Illustration of CO_2_‐EOR process using WAG injection technique. Reproduced under terms of the CC‐BY license.^[^ ^131^ ^]^ Copyright 2018, Springer Nature.) e) Comparison of the effect of CO_2_‐responsive compound ODPTA with conventional surfactants, such as SDS and octadearyl dimethyl ammonium chloride (OTAC). Reproduced with permission.^[^ ^132^ ^]^ Copyright 2017, Elsevier.

Self‐assembly structures are also promising candidates to construct switchable viscosifiers in an aqueous solution. Worm‐like micelles (WLMs) are the elongated, flexible self‐assemblies formed by the aggregation of amphiphiles,^[^ ^127^ ^]^ which exhibit remarkable viscoelastic properties above threshold concentrations. Moreover, the dynamic structures of WLMs can be influenced by external conditions (stimuli), whereas such morphology changes are usually reversible. Akbulut and his co‐workers reported the use of a pH‐responsive amphiphilic system in EOR.^[^ ^106^ ^]^ WLMs were assembled by the combination of an amino amide (*N*‐oleicamidopropyl‐*N,N*‐dimethylamine) and maleic acid (**Figure** **10**a), which can increase its viscosity 12 times by changing the pH from 4 to 8 in a reversible manner (Figure 10b). WLMs were proved to be effective as the displacing fluid in column experiments (Figure 10c). The same group also proposed a similar approach using a thermal‐responsive amphiphile with a WLMs formation as well as good salinity tolerance.^[^ ^128^ ^]^ Since the in situ EOR is usually operated at the elevated temperature, responsive amphiphiles could be injected on the ground at ambient temperature and self‐adapted to high viscosity according to the environment temperature.

#### 3.3.2 Switchable Self‐Assembled Viscosifier for CO_2_‐EOR

In the oil reservoirs with ultra‐low permeability, the EOR process using CO_2_ flooding (CO_2_‐EOR) is one of the most promising techniques to improve sweep efficiency. However, the high mobility of CO_2_ and the reservoir heterogeneity are considered the two major issues needed to be tackled.^[^ ^129^ ^]^ CO_2_ has a low density and high mobility. Thereby, CO_2_ gas tend to migrate either upward to the top of the reservoir or sideways to the regions with relatively higher permeability, both of which can be prevented by controlling system viscosity. Since CO_2_ gas is already involved in the process, it is quite intuitive to design CO_2_‐switchable viscosifiers to alleviate the above‐mentioned concerns. Li et al. investigated the use of a CO_2_‐responsive chemical (*N*‐erucamidopropyl‐*N,N*‐dimethylamine) for mobility control in the CO_2_‐EOR process using water‐alternating‐gas (WAG) injection.^[^ ^130^ ^]^ During the WAG process, water and gas (CO_2_) are injected into the wellbore alternatively, which provides a natural platform for the responsive viscosifier to be switched reversibly (Figure 10d).^[^ ^131^ ^]^ In an ideal scenario, the fluid viscosity maintains a low value before the contact with CO_2_ and exhibits a sharp increase by forming WLMs structures after the contact with CO_2_. Later on, the same group further improved the concept by screening more CO_2_‐responsive compounds at high temperature, high pressure, and high salinity conditions, which simulates the in situ operation.^[^ ^132^ ^]^ The long‐chain polyamine, ODPTA (octadecyl dipropylene triamine), displayed the most promising mobility control capability in the sand‐pack flooding experiments compared with conventional surfactants (Figure 10e). More importantly, ODPTA exhibited extraordinary CO_2_ foaming capability at a high temperature (160 °C), high pressure (7.8 MPa), and high salinity (200 000 ppm with 1000 ppm Ca^2+^). Similarly, Zhang et al. screened different types of chemicals with various CO_2_‐responsive groups for the CO_2_‐EOR process combined with hydraulic fracturing.^[^ ^133^ ^]^ Yang and his co‐workers also claimed that CO_2_‐responsive WLMs formed by the combination of sodium dodecyl sulfate (SDS) and diethylenetriamine (DETA) could enhance the performance of CO_2_ flooding.^[^ ^134^ ^]^ In general, switching the fluid viscosity in situ has a significant impact on improving oil recovery and broadening reservoir processability. WLMs that contain CO_2_‐switchable moieties are believed to have a good affinity with the CO_2_‐EOR process.

Although numerous switchable WLMs had been reported in various oil recovery‐related applications, as discussed above, there are still concerns about the feasibility of WLMs assemblies in harsh EOR operating conditions. Compared to polymers, WLMs feature dynamic assembly properties through non‐covalent interactions. Although assembling WLMs structure is much easier than the polymerization process, as well as that the non‐covalent bonds provide the accessible sites to design responsive behaviors, WLMs are difficult to maintain stable physicochemical properties when environmental changes lead to disruption. The viscoelastic property of WLMs solutions could be significantly reduced in the presence of light crude oil due to the transformation from WLMs to spherical micelles.^[^ ^135^ ^]^ Also, there is still a lack of evidence as to whether WLMs will retain their entangled structures when passing through a porous medium. The mechanical strength of WLMs could be challenged by the pore throats, where the capillary pressure becomes extremely high, according to Equation (2). To alleviate such concerns, Zhu et al. reported that adding a pre‐flush slug or post‐flush of HPAM would remarkably increase the oil recovery factor from the WLMs‐assisted process.^[^ ^136^ ^]^ To the best of our knowledge, however, there has been no further investigation of this system in switchable WLMs systems. Furthermore, economic efficiency is perhaps the biggest limitation to commercialize WLMs in EOR applications. The formation of WLMs requires the high concentration of the amphiphiles, typically above 4 wt%,^[^ ^137^ ^]^ which is not viable in large‐scale applications. In addition to a high chemical dosage, the adsorption onto solid surfaces could further consume the amphiphiles, leading to less efficiency of WLMs formation, lower fluid viscosity, and even the transformation from WLMs to spherical micelles.^[^ ^135a^ ^]^ There are emerging demands to develop the building blocks for WLMs with weak adsorption characters. Despite the challenges in the current technology, we believe that responsive WLMs still provide great opportunities for EOR applications.

## 4 Switchable Solvent

### 4.1 Role of Solvent

The use of solvents is prominent in numerous processes in the industry, including oil recovery, refinery, and oily waste cleanup. In these processes, solvents are mostly introduced to dissolve and mobilize heavy oils, as they have extensive influences on viscosity control, solid/liquid separation, and the removal of product impurities.^[^ ^3b^ ^]^ However, any process that involves the use of solvents is relatively expensive, not only due to the purchase, storage, use, and recovery of valuable solvent, but also because of the increased cost for operation safety and risk management.^[^ ^138^ ^]^ Hence, it is critically important to find an effective solvent that demonstrates good solubility with the target oils at a low solvent dosage.

The Hildebrand solubility parameter (*δ*), initially proposed by Joel Hildebrand in 1936, is commonly applied to estimate the ability of heavy oil dissolution in non‐polar solvents. The Hildebrand solubility is defined by the following equation^[^ ^139^ ^]^

(11) $$\delta ={\left(E_{\mathrm{co}}\right)}^{1/2}=\sqrt{\left(\Delta H_{\mathrm{vap}}-RT\right)/V_{m}}$$

where *E*_co_ is the cohesive energy density, Δ*H*_vap_ is the heat of vaporization, *R* is the gas constant, *T* is the absolute temperature, and *V*_m_ is the molecular volume. Hildebrand suggested that in order to dissolve a solute, which could be considered as a solvent molecule substituted by a solute molecule, the solute molecule has to overcome the intermolecular attractions between the solvent molecules.^[^ ^139^, ^140^ ^]^ The energy needed for solvency behavior is analogous to the evaporation of the solvent (Δ*H*_vap_) but excludes the pressure‐volume work (Δ*W* = *P*Δ*V*; for an ideal gas, *PV*/*n* = *RT*). Therefore, liquids with similar solubility parameters are more likely to interact with each other with fewer energy differences, resulting in better solvation and miscibility. The Hildebrand solubility parameter provides a satisfactory estimation of solubility for non‐polar and slightly polar systems, especially when no hydrogen bond is present. Efforts are also being made to extend the theory of the Hildebrand solubility parameter or to specify its application areas.^[^ ^141^ ^]^

The Hansen solubility parameter is another powerful tool to judge the solubility of heavy oil in a more complex system involving the contribution of hydrogen bonds. The Hansen solubility parameter consists of the energy from dispersion forces (*δ*_d_), dipolar intermolecular forces (*δ*_p_), and intermolecular hydrogen bonds (*δ*_h_). Hansen suggested that these three parameters can be imagined as the coordinates for a point in a 3D space, which is known as the Hansen space.^[^ ^142^ ^]^ Molecules that are closer to each other in the Hansen space are more likely to be compatible with each other. Typically, a value called the interaction (*R*_0_) radius is given to the solute (A), whereas the distance (*R*_a_) between the solute and solvent (B) and the relative energy difference (*RED*) indicator can be calculated by the following equations^[^ ^3^, ^142^ ^]^

(12) $$R_{a}^{2}=4{\left(\delta_{\mathrm{dB}}-\delta_{\mathrm{dA}}\right)}^{2}+{\left(\delta_{\mathrm{pB}}-\delta_{\mathrm{pA}}\right)}^{2}+{\left(\delta_{\mathrm{hB}}-\delta_{\mathrm{hA}}\right)}^{2}$$

(13) $$RED=R_{a}/R_{0}$$

The molecules (A and B) are likely to dissolve into each other if *RED* < 1, while the system is not miscible if *RED* > 1.

The solvent diffusion rate is another key parameter for the solvent‐involved processes. A faster diffusion rate means that the solvent can diffuse and penetrate into the heavy oil body more efficiently, resulting in a shorter time to generate the homogeneous solvent‐heavy oil mixture. The procedure of solvent diffusion into heavy oil is described by Fick's law of diffusion:^[^ ^143^ ^]^

(14) $$J_{i}=-D\cdot \frac{\partial c}{\partial x_{i}}$$

where *J*_i_ is the mass diffusion flux, *D* is the mass diffusion coefficient, and ∂*c*/∂*x*_i_ is the concentration gradient of the heavy oil in the solvent. Cormack et al. analyzed nine different solvents with various aromaticity and demonstrated that highly aromatic solvents, such as toluene, allow bitumen dissolution three to five times faster than a common aliphatic solvent, such as kerosene.^[^ ^144^ ^]^ On the other hand, Fu and Phillips reported that the diffusivity of solvent in heavy oil becomes faster with a decreased molecular weight of the solvent (*D*_pentane_ = 14.5 × 10^8^ cm^2^ s^−1^, *D*_hexance_ = 10.7 × 10^8^ cm^2^ s^−1^), whereas increasing aromatic content does not necessarily affect the diffusion coefficient (*D*_toluene_ = 7.78 × 10^8^ cm^2^ s^−1^, *D*_benzene_ = 8.19 × 10^8^ cm^2^ s^−1^).^[^ ^145^ ^]^ More recently, Chakrabarty et al. patented a solvent mixture (30 vol% of acetone and 70 vol% of pentane) for the best performance of oil sands extraction.^[^ ^146^ ^]^

**Scheme 3:**
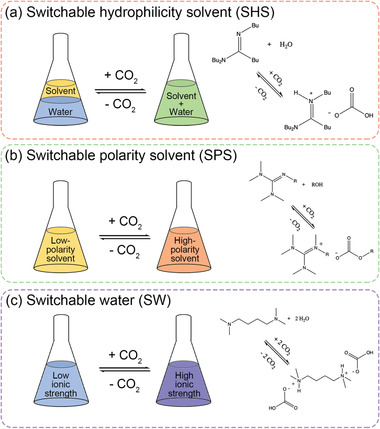
Three different types of switchable solvents and their switching mechanisms: a) switchable hydrophilicity solvent (SHS); b) switchable polarity solvent (SPS); and c) switchable water (SW).

In addition to seeking a solvent with a proper diffusion rate and solubility, growing attention has recently been paid to solvent toxicity and sustainability. There are two important reasons for this. First, increasing concerns about solvent emissions have led to stricter government and/or industrial regulations. Second, green solvents are being encouraged as a sustainable alternative to traditional solvents. Green solvents are defined by their benign properties, which pose little or no impact on the environment and human health. Obviously, water is the greenest solvent.^[^ ^147^ ^]^ Recent progress in the development of green solvents includes renewable solvents, ionic liquids, deep eutectic solvents (DES), liquid polymers, supercritical CO_2_ (scCO_2_), and switchable solvents. Due to our specific interests of switchable materials in this literature review, we will be focusing on switchable solvents that can be classified into three general categories (**Scheme** **3**): switchable hydrophilicity solvent (SHS), switchable polarity solvent (SPS), and switchable water (SW). Each category will be introduced in detail in the following sections.

### 4.2 Switchable Hydrophilicity Solvents

#### 4.2.1 SHSs for Oil Recovery

Solvent extraction is a promising technique for oil recovery due to its low operating temperature, high extraction rate, and universal applicability. However, traditional solvents are usually challenged by the costs of solvent recovery from the end product (e.g., distillation) and/or the inevitable solvent loss.^[^ ^3c^ ^]^ More recently, SHSs have been investigated as green substitutions for the traditional solvents. SHSs in the form of non‐ionic liquids (low polarity, water‐immiscible) can be converted to ionic liquids (high polarity, water‐miscible) when exposed to certain external stimuli, as illustrated in Scheme 3a.^[^ ^148^ ^]^ The most widely implemented stimulus for activating SHSs switchability is the CO_2_/N_2_ gas. SHSs in the form of ionic liquids feature low volatility, which greatly eliminates solvent loss due to evaporation. More importantly, their switchable character permits efficient recycling of the solvents from an organic mixture without an energy‐intensive distillation process, which makes SHSs distinct from the traditional solvents.

**Figure 11:**
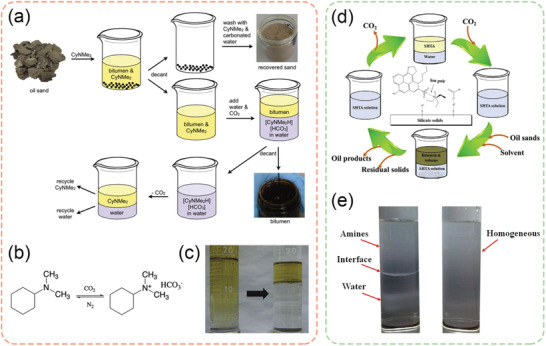
a) Proposed process of SHSs introduced as the non‐ionic liquid for bitumen recovery from oil sands. b) Mechanism of the CO_2_ switching solvent. c) Mixture of carbonated water and CyNMe_2_ after the bitumen has been decanted (left) and the same mixture after the CO_2_ has been removed (right). a–c) Reproduced with permission.^[^ ^149^ ^]^ Copyright 2012, Canadian Science Publishing.) d) SHSs introduced as ionic liquid for the enhancement of oil sands extraction. e) Amine‐water system before CO_2_ injection (left) and forming homogeneous solution after CO_2_ injection (right). d–e) Reproduced with permission.^[^ ^152^ ^]^ Copyright 2016, Elsevier.

SHSs can be introduced in their non‐ionic form to dissolve and dilute hydrocarbon resources. Once an SHS‐diluted oil stream is obtained, the SHSs can be switched to their ionic liquid form. They then become immiscible with hydrocarbons and phase‐separated from the high‐quality oil product for easy recycling. Holland et al. first investigated the use of an SHS (cyclohexyldimethylamine, CyNMe_2_) for enhancing bitumen recovery from oil sands.^[^ ^149^ ^]^ Their proposed process is illustrated in **Figure** **11**a–c. The SHS was deployed as a favorable solvent for the dissolving bitumen contained in the oil sands ores. After the filtration of the bitumen‐deprived sands, the liquid mixture of CyNMe_2_ and bitumen product could be easily separated by adding water and bubbling CO_2_, which switches the SHS from its nonpolar state to polar state. Consequently, the SHS with increased hydrophilicity became miscible with water and thereby migrated into the aqueous phase, whereas the bitumen retaining high hydrophobicity was phase‐separated spontaneously. Finally, the nonpolar‐state SHS could be restored by removing CO_2_ in the aqueous phase through N_2_/air purging, which allows the convenient recycling of the SHS for the next use. It should be mentioned that in this process, water was not involved in the bitumen extraction from oil sands until it was used for solvent recycling. Therefore, such an operation should be considered as a pure solvent extraction process. Similar strategies have been applied to various non‐conventional oil resources with recovery ratios typically higher than 90%.^[^ ^149^, ^150^ ^]^ In addition, SHSs have also been shown to weaken asphaltene self‐association interactions and reduce the size of asphaltene aggregations,^[^ ^151^ ^]^ both of which have positive implications on downstream operations.

However, similar to traditional solvents, the solvent loss is still the biggest challenge for commercializing SHSs in the solvent extraction process from the perspectives of both environmental regulators and industry. Although the switchable solvents supposedly used a straightforward recovery method to recycle solvents, to the best of our knowledge, the solvent loss still failed to meet most of the requirements. Holland et al. indicated that the best method applied resulted in a solvent loss of 0.06 g CyNMe_2_ per gram of solvent‐free bitumen.^[^ ^149^ ^]^ Merchan‐Arenas reported that only 54–60% of the SHS (*N,N*‐dimethyl‐cyclohexylamine, DMCHA) could be recycled for the next extraction process.^[^ ^150a^ ^]^ Such an issue is most likely to be caused by the insufficient protonation/deprotonation of SHSs during the CO_2_ switching process, which leads to incomplete phase separation of SHSs from hydrocarbon products. On the other hand, the low mobility of the oil resources with higher viscosity (such as heavy oil and bitumen) is also considered a potential hindrance to the recovery of the switchable solvent.^[^ ^3c^ ^]^

SHSs can also be introduced in their ionic form, typically in combination with the aqueous phase, where they serve as both the solvent and the interfacial modifier. Such a strategy is typically referred to as a solvent‐assisted ambient aqueous hybrid extraction (SA^3^HE). Sui et al. reported the use of a switchable‐hydrophilicity tertiary amine (SHTA) in recovering heavy hydrocarbon from oil sands (Figure 11d,e).^[^ ^152^ ^]^ A cosolvent (toluene) was added to further reduce the bitumen viscosity. It was inferred that the processability was improved due to the formation of ion pairs at the bitumen surfaces, which inhibited the bitumen‐solid interactions. Sui's group later claimed a similar approach using a CO_2_ switchable solvent with a diamine structure (*N,N,N′,N′‐*tetraethyl‐1,3‐propanediamine, TEPDA), but in the absence of the cosolvent.^[^ ^153^ ^]^ In this study, SHSs was demonstrated with promising recyclability and a significant reduction in solid entrainment in the hydrocarbon product. Switchable solvents combined with the aqueous phase could be recycled at least four times and only exhibited a slight decrease in extraction efficiency.^[^ ^152^, ^153^ ^]^ Additionally, Li et al. emphasized that both the solid entrainment in the oil product and the solvent loss to solid wastes were sharply reduced (more than 50%).^[^ ^153^ ^]^ Once again, such a phenomenon should be attributed to the decreased interaction between bitumen and the solids. However, the major concern of applying ionic SHSs is also originated from their interaction with solids. Since the approach of combining SHSs with water is highly dependent on the replacement of oil‐solid interactions by oil‐solvent ion pairs, using SHSs in ionic liquid form might be significantly highly affected by the solid properties, as well as by the affinity between bitumen and switchable solvents. On the other hand, the physical and chemical properties of the reservoir rock are often location dependent. Hence, the strategy of applying SHSs in their ionic form may not be as universally applicable as the non‐ionic switchable solvent extraction. Further exploration is needed to investigate the feasibility of switchable solvents in various oil resources, especially when the host solids are partially hydrophobic.

#### 4.2.2 SHSs for Oily Waste Cleanup

**Figure 12:**
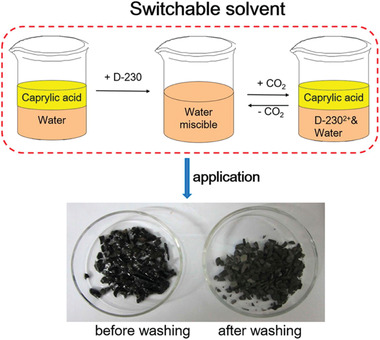
Top: CO_2_‐switchable phase behavior of the SHS formed by Jeffamine D230 and caprylic acid (C8). Bottom: Washing efficiency of the D230‐C8 SHS on oily drilling cut. Reproduced with permission.^[^ ^10e^ ^]^ Copyright 2017, Elsevier.

SHSs are also being studied for remediation of oil‐contaminated solids. Chen et al. developed an SHS system that is based on the ion‐pair interactions between medium‐chain fatty acids (MCFAs) and poly(oxypropylene) diamine (Jeffamine D230).^[^ ^10e^ ^]^ MCFAs were selected as the building block for designing SHSs because they are biocompatible and abundant in nature. MCFAs are naturally immiscible with water in their carboxylic acid forms but become water‐soluble when combined with proper amines (e.g., Jeffamine D230). The liquid mixture was applied as the washing fluid to remove the oily contaminants in the drilling cut, whereas the effluents could be treated with CO_2_ bubbling to separate the process water (**Figure** **12**). More importantly, both Jeffamine D230 and fatty acid show negligible adsorption on solid surfaces, which is essential to avoid secondary contamination.

### 4.3 Switchable Polarity Solvents

SPSs are low polarity solvents that can switch to their ionic‐liquid form (high polarity) in the presence of CO_2_ (Scheme 3b). Compared with SHSs, SPSs do not contain a biphasic stage. Instead, they change polarity as an organic solvent. Typically, SPSs are composed of a nucleophilic solvent, such as alcohols or amines, and an organic base, such as amines, amidines, or guanidines.^[^ ^154^ ^]^ The nucleophilic agent (alcohols or amines) can chemically bond with CO_2_ gas and transform into ammonium carbonate or carbamate anions,^[^ ^154d^ ^]^ both of which can form ion pairs with the organic base and result in the increase of solvent polarity. Such a process is fully reversible by heating and/or purging of inert gas. SPSs have been shown to be a good choice of green solvent for various organic synthesis processes.^[^ ^155^ ^]^ There are also plenty of investigations into using SPSs in applications such as post‐combustion CO_2_ capture,^[^ ^156^ ^]^ soybean oil extraction,^[^ ^157^ ^]^ heavy metals extraction,^[^ ^158^ ^]^ and lignocellulose pretreatment.^[^ ^159^ ^]^ Compared with SHSs, however, SPSs have been neither investigated in applications of either oil recovery or oily waste cleaning up. The biggest limitation of SPSs in these fields is their vulnerability to water. Specifically, in the presence of water, the organic bases (amines, amidines, and guanidines) tend to form bicarbonate salts first, which is more thermodynamically stable than carbonate or carbamate salts.^[^ ^160^ ^]^ As a result, a strict drying process is required before activating the switching behavior of SPSs. Also, the range of polarity switching is relatively moderate in SPSs compared to that in SHSs.^[^ ^148^ ^]^ Although such a small change could cause significant differences in many applications, it is most likely to be insufficient in either oil recovery or oily waste cleanup applications, especially when combined with SPS's water sensitivity.

### 4.4 Switchable Waters

**Figure 13:**
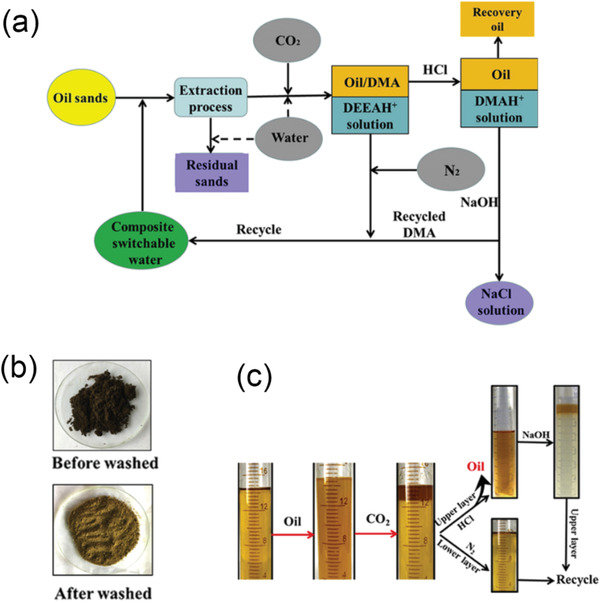
a) Proposed process of washing oil sands with switchable water (SW) (DMA+DEEA (aq)). b) Oil sands before and after being washed with the SW. c) Process of separating simulated oil from the oil‐SW mixture and recycling of the SW composites. Reproduced with permission.^[^ ^162^ ^]^ Copyright 2018, Elsevier.

SWs are aqueous solutions that can switch from low ionic strength to high ionic strength reversibly (Scheme 3c). The switching mechanism of SWs is typically based on an amine component, which forms bicarbonate salt in the presence of CO_2_.^[^ ^161^ ^]^ Compared to SHSs, SWs are miscible with water both before and after switching. Recently, Li et al. developed a new process for oil sands washing using the SW composited by *N,N*‐diethylethanolamine (DEEA) and *N,N*‐dimethylaniline (DMA).^[^ ^162^ ^]^ DEEA was screened from five different types of amines and was demonstrated to have excellent CO_2_ responsiveness due to the existence of two electron‐donating groups (ethyl groups), which increase the electron density of the nitrogen atom and enhances its activity.^[^ ^162^, ^163^ ^]^ Meanwhile, a hydrophobic tertiary amine was applied to enhance the maximum dissolving amount of DEEA in solutions. DMA was selected as the most suitable candidate as it has a steadier molecular state than DEEA at the same pH condition. The proposed washing procedure is illustrated in **Figure** **13**a. In general, simulated oil sands samples were mixed with the SW (DMA+DEEA, the ratio of DMA in grams to DEEA solutions in mLs was 4:15) to extract the valuable oil products. The percentage of oil removal reached 90.24 wt%, which was also confirmed by the clean sands after washing (Figure 13b). The oil content of the residual sands reached 0.872 wt%, which meets most of the regulations for safe disposal. After the extraction, DEEA was recycled as DEEAH^+^ through CO_2_ bubbling. The recycling of DMA requires a deeper degree of protonation using an HCl solution (Figure 13c), giving a DMA recovery of 78.31 wt%. The authors claimed that such high solvent loss was most likely caused by the heat that was generated during HCl and NaOH addition. Apart from the applications in oil recovery, SWs have also been investigated for CO_2_ capture,^[^ ^164^ ^]^ as well as the removal of water‐soluble organics and/or clays throughout the salting‐out effect.^[^ ^161^, ^165^ ^]^

## 5 Switchable Wettability

### 5.1 Role of Wettability

**Scheme 4:**
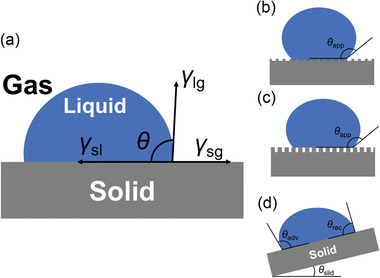
Schematic illustration of a liquid droplet on a solid with a smooth or rough surface (in gas). a) Young's model; b) Wenzel's model; c) Cassie‐Baxter's model; d) Measurement of sliding angle, advancing contact angle, and receding contact angle.

Surface wettability is an intrinsic property of a solid surface, which depends on the chemical composition and local topology.^[^ ^4^, ^23^, ^166^ ^]^ For the reader's convenience, a simple method to describe the wetting behaviors is to imagine a liquid droplet sitting on a solid surface surrounded by a gas environment (**Scheme** **4**a). The surface wettability of the solid could thus be described by the contact angle (*θ*). When considering the force balance acting on the three‐phase contact line at an equilibrium state, and assuming that the solid surface is perfectly flat, smooth, and chemically homogeneous, one can easily derive the following equation^[^ ^96^ ^]^

(15) $$\cos\theta =\left(\gamma_{\mathrm{sg}}-\gamma_{\mathrm{sl}}\right)/\gamma_{\mathrm{lg}}$$

where *γ*_sg_, *γ*_sl_, and *γ*_lg_ are the IFTs of solid‐gas, solid‐liquid, and liquid‐gas interfaces, respectively. Equation (15) is also known as Young's equation.

In the real world, a solid surface is not always ideal. A solid surface often contains topological structures and/or compositional heterogeneity. Both of the two factors could affect the apparent contact angle values significantly. In 1936, Wenzel considered the roughness of a solid surface and assumed that the size of the roughness is sufficiently small, as illustrated in Scheme 4b.^[^ ^167^ ^]^ In Wenzel's model, the apparent contact angle (*θ*_app_) on a “real” surface is related to the contact angle on an ideal solid surface by the following equation:^[^ ^167a^ ^]^

(16) $$\cos\theta_{\mathrm{app}}=\;r\cos\theta$$

where *r* is the rugosity/roughness factor. This roughness factor is defined as the ratio of the geometrically projected surface area to the “smooth” area (*r* = *A*/*A*_smooth_). From this definition, it can easily be seen that *r* ≥ 1, since the “smooth” area is always a part of the total projected area. Therefore, Wenzel's model shows that the roughening of a smooth surface always magnifies the current state of the surface wettability. Namely, if a smooth surface has a contact angle less than 90°, it will exhibit an even lower apparent contact angle with the presence of surface roughness. On the other hand, a hydrophobic surface (*θ* > 90°) will show a larger apparent contact angle when counting the surface roughness. Such effects are more significant in the case of a higher value of “*r*”, i.e., a rougher surface.

Another approach for the interpretation of surface roughness was given by Cassie and Baxter.^[^ ^168^ ^]^ They assumed that a rough surface could be considered as a surface composed of two different types of patches, of which the intrinsic contact angles are *θ*_1_ and *θ*_2_, respectively. In the Cassie‐Baxter model, the apparent contact angle can be written as the following^[^ ^168^ ^]^

(17) $$\cos\theta_{\mathrm{app}}=\phi_{1}\;\cos\theta_{1}+\phi_{2}\cos\theta_{2}$$

where *φ*_1_ and *φ*_2_ are the area fractions for the two types of patches, respectively. One of the most important examples is that gas (e.g., air) can be trapped inside the surface roughness, where the liquid (e.g., water) does not touch the solid in these regions, as illustrated in Scheme 4c. If assuming a 180° contact angle of the liquid to air, Equation (17) could be rewritten as the following

(18) $$\cos\theta_{\mathrm{app}}=\phi_{1}\cos\theta +\left(1-\phi_{1}\right)\cos\pi =\phi_{1}\cos\theta +\phi_{1}-1$$

where *φ*_1_ represents the area fraction of the liquid coming into contact with the solid surface. Equation (18) indicates that the apparent contact angle of an air‐trapped rough surface can become closer to 180°, if the solid area fraction (*φ*_1_) is sufficiently small. For example, a water drop sitting on a lotus leaf only has about 2–3% contact area with the leaf surface, which yields a contact angle of 180° under high resolution confocal microscopy.^[^ ^169^ ^]^

However, it has to be denoted that the measurement of the static contact angle often contains optical artifacts, especially for the surfaces with microscopic roughness. The apparent contact angle measured on such surfaces is typically not the actual equilibrium contact angle. Moreover, the apparent contact angle could even be different between the droplets being deposited onto the surface or being created at the surface.^[^ ^170^ ^]^ Apart from the static contact angle, surface wettability could also be described by various parameters, includes the sliding angle (also known as the roll‐off angle) and the contact angle hysteresis. The sliding angle (*θ*_slide_) is the angle of surface inclination when a water droplet is able to roll off completely from a tilted surface without external forces other than gravity (Scheme 4d). Sliding angle represents the droplet retention force throughout the following equation:

(19) $$F_{\mathrm{re}}=mg\sin\theta_{\mathrm{slide}}$$

where *F*_re_ is the retention force on an inclined surface. It is obvious that a lower sliding angle refers to a weaker retention force, and an easier roll‐off of the droplet. For example, the lotus leaf exhibits a water sliding angle lower than 5°.^[^ ^171^ ^]^ When the droplet is rolling on the inclined surface, the contact angle measured at the front side (advancing contact angle, *θ*_adv_) and that at the tail end (receding contact angle, *θ*_rec_) are not the same, as illustrated in Scheme 4d. The deviation between the values of *θ*_adv_ and *θ*_rec_ is known as the contact angle hysteresis (*θ*_adv_ – *θ*_rec_), which is also originated from the retention force. Therefore, the contact angle hysteresis is typically smaller for surfaces with smaller sliding angle. Obviously, both sliding angle and contact angle hysteresis could provide useful information on determining the properties of surfaces with very high static contact angle, such as superhydrophobic surfaces.^[^ ^170^, ^172^ ^]^

Owing to the development of fundamental understandings of surface wettability, special wettable surfaces have been fabricated with advanced wetting properties. For example, a superhydrophobic surface (ultrahigh water contact angle in the air) can be designed by choosing the materials with low surface energy (*γ*_sg_) and then increasing the surface roughness. There is an increasing amount of research on the fabrication and application of special wettable materials. Readers interested in learning more can refer to the reviews written by Xue et al.^[^ ^166^ ^]^ and Li et al.^[^ ^173^ ^]^

More recently, special wettable materials with on‐demand switchable wettability have attracted considerable attentions as the cutting‐edge technology for the oil spills cleanup and oily wastewater treatment. More specifically, responsive absorbents have been designed with oil‐absorbing characteristics by introducing superhydrophobic and superoleophilic surface. These responsive absorbents could be switched to the opposite conditions for spontaneous oil desorption and self‐cleaning purposes. In the following sections, we will discuss the strategies used for designing these novel responsive materials and the most recent developments in designated area.

### 5.2 Responsive Absorbents

Oil spills that release toxic petroleum hydrocarbons are among the most significant threats to ocean and coastal ecosystems. Containing oil spills requires restricting the spread and removing the oily pollutants in the first stage of the spill to minimize the consequences. Typical methods for an oil cleanup involve controlled in situ burning,^[^ ^174^ ^]^ use of dispersants,^[^ ^175^ ^]^ or absorbents,^[^ ^4^, ^176^ ^]^ mechanical recovery (blooms, skimmers, or vessels),^[^ ^177^ ^]^ and bioremediation.^[^ ^178^ ^]^ However, some of these methods have been known to cause severe secondary pollutions, e.g., air pollution from in situ burning and algal blooms (also known as the “red tides”) caused by dispersants.^[^ ^4^, ^179^ ^]^ More recently, the use of absorbents has been considered an effective strategy since they have the potential to clean up the oil spills efficiently, contain the oils safely in a semi‐solid state, and recover the valuable hydrocarbon resources.

**Scheme 5:**
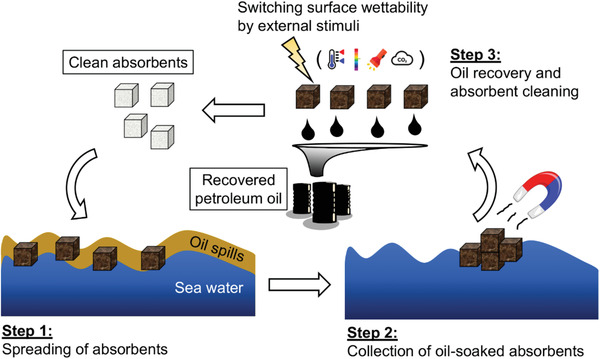
A typical process of oil spill cleanup, hydrocarbon recovery, and absorbents recycling. The roles of novel responsive absorbents with magnetic‐responsiveness and/or switchable surface wettability are indicated at their corresponding steps.

There are typically three major steps when using absorbents for an oil spills cleanup, as illustrated in **Scheme** **5**. Initially, a large quantity of absorbents is spread over the polluting site to absorb the oil spills (Step 1). Absorbents are typically made of materials with a high surface area and porous structure, including inorganic porous materials,^[^ ^180^ ^]^ organic synthesized porous materials,^[^ ^9^, ^181^ ^]^ and biomaterials.^[^ ^182^ ^]^ Although these prototype materials are very cheap and easily accessible, they often contain intrinsic water affinity, which limits their oil absorption capacity and floatability. Recent advances in surface science and nanotechnology have led to the fabrication of novel special wettable absorbents. These absorbents feature a specific micro‐/nano‐architecture, which results in surface superhydrophobicity and superoleophilicity, making it possible for the oily pollutants to be selectively absorbed.^[^ ^4^, ^183^ ^]^ Once the oils are completely absorbed, the absorbents need to be collected (Step 2). Absorbents with the magnetic‐responsive feature are believed to possess significant advantages at this stage since their movements can be easily directed by an external magnetic field.^[^ ^184^ ^]^ In the final stage, it is desirable to recover the valuable oils from the absorbents (Step 3). However, the traditional method of mechanical squeezing is only applicable to elastic materials and often leads to an incomplete oil removal due to the oleophilic nature of the absorbent surfaces. On the other hand, the commonly applied recovery process of distillation requires extra energy investment and also accompanies solvent loss issues. Stimuli‐responsive absorbents (StiRAs) offer a new way to achieve efficient oil removal by introducing switchable surface wettability that can transform from superoleophilic to superoleophobic. Such a transition makes the oil desorption spontaneously occur, thereby easily recovering the oils from the StiRAs and leaving the clean materials for the next use. In the following paragraphs, we will discuss the recent progress in designing both magnetic‐responsive absorbents (MagRAs) in the absorbents collection step, as well as StiRAs with switchable surface wettability for the oil desorption stage.

#### 5.2.1 Magnetic‐Responsive Absorbents (MagRAs) for Absorbent Collection

**Figure 14:**
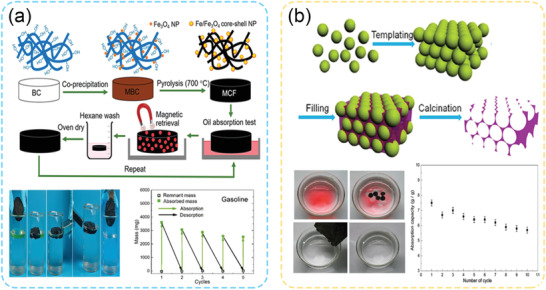
a) Top: Synthesis routine of magnetic carbon fiber (MCF) aerogels and their proposed functions in the oil absorption process. Bottom: Absorption of “green oil spills” floating on water using an MCF aerogel, and the recyclability of the MCF aerogel by dissolving in hexane after oil absorption. Reproduced with permission.^[^ ^186^ ^]^ Copyright 2020, American Chemical Society. b) Top: Synthesis of 3D macro‐porous Fe/C nanocomposites. Bottom: Removal of lubricant oil (red) from water and the recyclability of the Fe/C nanocomposite over 10 cycles. Reproduced with permission.^[^ ^188^ ^]^ Copyright 2012, American Chemical Society.

In the past decade, MagRAs have attracted special recognition in the field of designing “intelligent” absorbents. These novel materials can be easily directed to the oil spill location and collected through the guidance of an external magnetic field. Their well‐controlled motions are especially meaningful in the marine environment, where oil‐soaked absorbents might be blown away by the strong wind or water flows, leading to secondary pollutions.^[^ ^185^ ^]^ Typically, three different strategies can be utilized to fabricate novel MagRAs. Strategy (i) involves grafting of both hydrophobic moieties and magnetic materials onto a porous substrate. For example, Ieamviteevanich et al. incorporated magnetic NPs (Fe/Fe_3_O_4_ core–shell NPs) onto bacteria cellulose (BC)‐derived carbon nanofiber (CNF) aerogel (denoted as MCF in **Figure** **14**a).^[^ ^186^ ^]^ The fabricated MCF aerogel exhibited ultralow density (7.4 ± 1.2 mg cm^−3^), very high oil absorption capacity (37–87 g g^−1^), and strong saturation magnetization (≈102 emu g^−1^). Strategy (ii) of fabricating MagRAs involves the direct hydrophobization of a prepared magnetic porous material. Vivek and Prasad modified Fe_2_O_3_ with a poly(amidoamine)‐based dendrimer, poly(amidoamine)‐(3‐acryloyloxy)‐trimethoxysilane (PAMAMOS).^[^ ^187^ ^]^ The system not only removes engine oil from water at an absorption capacity of 22.9 g g^−1^, but can also be extended to form a self‐healing membrane for water desalination. Strategy (iii) is the direct mixing of hydrophobic and magnetic materials. Chu and Pan fabricated 3D Fe/C nanocomposites by the calcination process, utilizing polystyrene (PS) as the template (Figure 14b).^[^ ^188^ ^]^ The concentration of Fe and C could also be adjusted to modify the surface wettability and magnetic response in an effort to optimize oil removal and MagRAs recycling efficiency. In addition, to provide a comprehensive explanation of the current developments in this field, some recent publications of MagRAs are listed in **Table** **3**.

**Table 3:** Recent advances in high‐performance MagRAs for oil spills cleanup and magnet‐assisted absorbents collection

Strategy	Materials	WCA	Saturated absorption capacity [g g^−1^]	Saturation magnetization [emu g^−1^]	Ref.
(i)	ENR‐Fe_3_O_4_ (ENR: Epoxidized natural rubber)	–	Gasoline: 2.6–6.8	–	^[^ ^182a^ ^]^
	CNT‐Fe_3_O_4_ (CNT: Carbon nanotube)	–	Diesel oil: 6.6	≈15	^[^ ^192^ ^]^
	PU‐PTFE/Fe_3_O_4_ (PU: Vinyltrimethoxysilane; PTFE: Polytetrafluoroethylene)	>160°	Mineral oil: 13.25	–	^[^ ^181a^ ^]^
	PU‐Fe_3_O_4_‐FAS‐17	153.7°	Gasoline: 17.5 Lubricant oil: 12 Kerosene: 11.9 Petroleum ether: 11 Solvent naphtha: 10.5	–	^[^ ^193^ ^]^
	PDMS‐ZnFe_2_O_4_ silicone (PDMS: Polydimethylsiloxane)	146°	Paraffin oil: 4.44 Motor oil: 4.13 Silicone oil: ≈5	0.88 (ZnFe_2_O_4_ NP); 0.065 (Silicone).	^[^ ^190c^ ^]^
	Carbon sponge‐ferrocene	156.8°	Gasoline: 27 Petroleum ether: 17	7.5	^[^ ^194^ ^]^
	CMC/Na^+^‐MMT‐Fe_3_O_4_ aerogel (MMT: Montmorillonite)	121°	Pump oil: 19.2 Gasoline: 17.4 Corn oil: 15.6 Sesame oil: 14.3 Soybean oil: 13.1 Olive oil: 10.5	31.10	^[^ ^182b^ ^]^
	Pyrolyzed BC‐Fe/Fe_3_O_4_ NPs aerogel	107.2°	Gasoline: 67 Diesel oil: 60 Sunflower oil: 50 Soy oil: 40 Palm oil: 38 Coconut oil: 37	102	^[^ ^186^ ^]^
	Graphene‐Co‐resorcinol/formaldehyde composited aerogel	124°	Pump oil: 69 Soybean oil: 67 Gasoline: 60 Diesel oil: 58	18	^[^ ^190d^ ^]^
	Graphene‐Fe_3_O_4_‐PS composited aerogel	142.2°	Crude oil: 40 Lubricant oil: 37 Diesel oil: 30	With 50 wt% Fe_3_O_4_: ≈28; With 40 wt% Fe_3_O_4_: ≈20; With 12 wt% Fe_3_O_4_: ≈9.	^[^ ^191b^ ^]^
(ii)	Fe/popcorn‐OCS	151.6°	Corn oil: 10.28 Engine oil: 10.02	3.3 (Decrease with decreasing Fe(NO_3_)_3_ concentration; Increase with increasing carbonization temperature.)	^[^ ^182c^ ^]^
	Fe_3_O_4_@SiO_2_ NPs‐DMDEOS/VTMS (DMDEOS: Dimethyldiethoxysilane; VTMS: Vinyltrimethoxysilane)	161.5°	10.21–13.77	10.0	^[^ ^195^ ^]^
	Magnetic MWNT‐PDMS sponges (MWNT: Multi‐wall nanotube)	153.4°	Gasoline: 11.1 Petroleum ether: 8.8	–	^[^ ^196^ ^]^
	Fe_2_O_3_/C‐TCMS aerogel (TCMS: Methyltrichlorosilane)	152°	Bean oil: 102.6 Lubricant oil: 101 Crude oil: 89.3 Hexane: 61.8	–	^[^ ^180a^ ^]^
	Fe_2_O_3_‐PAMAMOS aerogel	91.34°	Engine oil: 22.9	68 (5 K); 61 (300 K).	^[^ ^187^ ^]^
	Fe_3_O_4_/Cellulose‐TiO_2_ aerogel	–	Paraffin oil: 28	–	^[^ ^189a^ ^]^
(iii)	3D Fe/C nanocomposites	> 152°	4.8–9.4	–	^[^ ^188^ ^]^
	Fe‐containing MOF (MOF: Metal–organic framework)	143°	Lubricant oil: 40 (Decrease to 30 after nine cycles)	26 (MOFs formed at 500 °C); 49 (MOFs formed at 600 °C).	^[^ ^191a^ ^]^
	PS/Fe_3_O_4_ NPs with hierarchical pore structure	133°	Dimethicone: 94 Edible oil: 87 Saxoline: 65	14	^[^ ^190b^ ^]^
	Fe/CNT sponges	145°	Diesel oil: 56 Gasoline: 49	21.1	^[^ ^197^ ^]^
	NCA/OA/ Fe_3_O_4_ aerogel (NCA: Nanocellulose aerogel)	84.5°	Pump oil: 33.24	–	^[^ ^189b^ ^]^

Three essential parameters are highlighted in the table for the evaluation and comparison of each MagRAs: water contact angle (WCA), saturated absorption capacity, and saturation magnetization. Most MagRAs feature high water contact angles (>140°), illustrating decent surface hydrophobicity. Although not shown in the table, the oil contact angles (OCAs) of these MagRAs are mostly zero, demonstrating the spontaneous spreading of the oils on these surfaces. Collectively, these materials would strongly repel the water while attracting to the spilled oil. Meanwhile, the saturated absorption capacity is calculated from the weight ratio of absorbed oil to the absorbent itself. Therefore, a higher value of saturated absorption capacity indicates that the corresponding MagRAs can carry more oils. For example, MagRAs in the form of aerogels usually possess a significant advantage from the perspectives of saturated absorption,^[^ ^180^, ^182^, ^186^, ^187^, ^189^ ^]^ which is attributed to their ultralight weight and ultralow density. It is also worth noting that the oil absorption capacity is closely related to oil density and viscosity. The high‐viscosity oils tend to block the porous materials when being absorbed due to their slow movements in the confined environment.^[^ ^190^ ^]^ Even worse, air can become trapped inside the porous medium, decreasing the “effective” surface area for oil absorption. Both of these issues suggest that it is harder to collect more viscous oil. Finally, the saturation magnetization is the indicator describing how MagRAs respond to the external magnetic field, which is the most attractive characteristic of these novel absorbents. The values of saturation magnetization are typically decreased with decreasing concentrations of the magnetic‐responsive content, e.g., Fe_3_O_4_.^[^ ^182^, ^191^ ^]^ MagRAs with a stronger magnetic response are preferable in actual scenarios since they are less likely to be lost in the water environment. Indeed, all of the MagRAs listed in Table 3 could easily be attracted by a permanent magnet in a laboratory environment.

Meanwhile, the application of MagRAs for oil spills treatment still suffers from several major concerns. When the oil spill occurs, it commonly impacts an extremely wide range of open water areas. However, the magnetic force decays rapidly with the distance to the magnet (*F* ∝ *r* ^−2^). This means that the difficulties in controlling the movement of MagRAs through the magnetic field would increase dramatically when the area of oil spills is large. Moreover, this challenge becomes even more severe, since the weight of MagRAs also increases after absorbing the oil spills. Future investigations of MagRAs are anticipated to explore more on the feasibility of these novel responsive absorbents in large‐scale implementations.

#### 5.2.2 Stimuli‐Responsive Absorbents for Oil Removing/Recycling

In the final stage of oil spill treatment, petroleum oils should be recovered from the absorbents to reimburse some of the cleaning costs, as well as to recycle the absorbents. However, oil desorption from traditional absorbents, which has relied on mechanical squeezing or distillation, is usually inefficient or energy‐consuming. Such difficulties are consequences of high oil adhesion characteristics of traditional absorbents, which are designed to absorb in the first stage of oil spills.^[^ ^198^ ^]^ Namely, traditional absorbents with better absorbing properties will experience more of a struggle at the oil desorption stage. Furthermore, these irremovable oil components can block up the oil‐absorbing tunnels, decrease the saturated absorption capacity, and eventually result in an oil‐fouling effect.^[^ ^182d^ ^]^

Effective absorptions followed by a complete recovery of the oils are a priority when designing novel absorbents.^[^ ^199^ ^]^ StiRAs are capable of switching their surface wettability from oleophilic to oleophobic when exposed to the desired external stimulus, such that the oil desorption process can be activated on‐demand. If the switching process is reversible, StiRAs can also be recycled by transforming back to their oleophilic state after oil removal. We listed some of the recent publications of StiRAs in **Table** **4** as a review of the current progress in this field.

**Table 4:** Recent advances in StiRAs with switchable surface wettability for oil spills cleanup followed by oil removing/recycling

		Switchable range (Before – After if applicable)	
Switching trigger	Materials	WCA	OCA/UWOCA	Saturated absorption capacity [g g^−1^]	Desorption efficiency [%]	Ref.
pH	3D porous GF‐ (P2VP‐b‐PHA) (PHA: Poly(hexadecyl acrylate); GF: Graphene foam)	152°	OCA: 0°	Crude oil: ≈60 Pump oil: ≈55 Diesel oil: ≈50 Gasoline: ≈40	≈95–99	^[^ ^199^ ^]^
	MF‐PVP sponge (MF: Melamine formaldehyde; PVP: Poly(4‐vinyl pyridine))	135°–0°	OCA: 0°; UWOCA: 0°–152°.	Dichloro‐methane: 49	0 (pH > 5); 40 (pH = 3); 100 (pH = 1).	^[^ ^200^ ^]^
	PU foam‐ (PDMA‐co‐PTMSPMA‐co‐PDEAEMA) (PTMSPMS: Poly(3‐trimethoxysilylpropyl methacrylate)	150°–0°	OCA: 0°; UWOCA: 0°–153°.	Diesel oil: ≈35 Gasoline: ≈32 Pump oil: ≈31 Petroleum ether: ≈25	Mostly released upon slight compression.	^[^ ^201^ ^]^
	Amine‐containing monoliths	129.2°–0°	UWOCA: 0°–147.8°	Pump oil: ≈10 Crude oil: ≈9.5 Edible oil: ≈9 Gasoline: ≈9	≈100 (pH = 1)	^[^ ^180c^ ^]^
	CF‐P4VP	155°–0°	OCA: 0° (pH = 7)–135° (pH = 1)	Crude oil: ≈71 Kerosene: ≈62	Crude oil: 83; Kerosene: 100.	^[^ ^202^ ^]^
pH and thermal	Cotton‐PDMAEMA (PDMAEMA: Poly(2‐dimethylaminoethyl methacrylate))	130°–0°	UWOCA: 0° (pH = 13)–130° (pH = 1)	Corn oil: ≈6 (pH = 1, 25 °C); ≈4.2 (pH = 13, 25 °C); ≈4.8 (pH = 6.5, 25 °C); ≈4 (pH = 6.5, 60 °C).	Desorption in cold acidic solutions; Anti‐oil‐fouling.	^[^ ^182d^ ^]^
Thermal	Regenerated cellulose (RC)‐PNIPAm nanofibers	113°–0°	OCA: 0°; UWOCA: 0°–160.2°	Silicone oil: 3.32 Paraffin oil: 3.18 Peanut oil: 2.95 Gasoline: 2.76	–	^[^ ^203^ ^]^
	MF‐OTS‐PNIPAm sponge (OTS: Octadecyltrichlorosilane)	150°–0°	UWOCA: 0°–148°	Peanut oil: ≈50 Pump oil: ≈46 Gasoline: ≈40 Petroleum ether: ≈35	At 20 °C: Large sponges exhibit slow desorption in 6 h; Small sponge pieces exhibit quick desorption in 135 s.	^[^ ^204^ ^]^
Light and thermal	M‐PNIPAm/PPy	143°–0°	OCA: 0° (40 °C); UWOCA: 157° (22 °C).	Bitumen: 5.85 Mineral oil: ≈5.43 Paraffin oil: ≈5.43 Silicone oil: ≈4.86 Crude oil: ≈4.57	Bitumen: >87; Crude oil: ≈91; Mineral oil: 91; Paraffin oil: ≈92; Silicone oil: ≈90. (Bitumen desorption decreased to 61% after 5 cycles.)	^[^ ^205^ ^]^
Light	Nano‐sponge composited of hydrocarbon NPs/TiO_2_ NPs (6:4)‐porous PDMS	140°–40°	OCA: 0°–0°; UWOCA: 125°–150°	Crude oil: 4.59	80 (Mechanical squeezing); 65 (48 h UV irradiation); 91 (1 h air bubbling); >98 (UV and air bubbling).	^[^ ^9b^ ^]^
	MF‐SPMA sponge (SPMA: Spiropyran‐containing methacrylate)	155.5°–27°	OCA: 0°	Silicone: ≈90 Pump oil: ≈85 Gasoline: ≈65	No oil desorption under visible light; High oil desorption (85%) under UV irradiation (365 nm) for 30 min.	^[^ ^206^ ^]^
CO_2_	PS‐ [1,4‐bis(diethyl‐amno)‐2,3‐bismethacryol‐oxybutanoate]	–	–	1.8	84.6 (upon CO_2_ stimulation)	^[^ ^207^ ^]^
Electric	Reduced graphene oxide (RGO)‐MS	131°	OCA: ≈27°–0°	–	3.87 g of crude oil was recycled by an in situ pumping process.	^[^ ^208^ ^]^
Plasma	Hyperbranched PU/Fluorine‐modified SiO_2_ NPs (PU: Polyurethane)	151°–0°	OCA: 0°; UWOCA: 152°.	Diesel oil: ≈20 Gasoline: ≈17.5	–	^[^ ^181b^ ^]^

Similar to the discussions for MagRAs, we highlight three primary parameters for the comparison of novel StiRAs materials: the switchable range of contact angles, saturated absorption capacity, and desorption efficiency. The contact angle switching range illustrates the change of surface wettability before and after the switch, which is one of the most interesting and important characteristics of the StiRAs. Ideally, the surface of StiRAs should be at an initial state of hydrophobic and oleophilic, which can be transformed to an opposite state (hydrophilic and oleophobic) after triggering the switch. In the table, the WCAs of StiRAs were typically very high (>140°), which switched to zero values by the stimulus. On the other hand, OCAs showed the opposite switching direction, which was turned from superoleophobic to superoleophilic. Clearly, a broader range of contact angle changes suggests a more significant difference before and after switching the wettability of StiRAs surfaces. From an application perspective, the saturated absorption capacity and the desorption efficiency can provide a more direct evaluation of StiRAs performances in the oil absorption‐desorption process. The former parameter demonstrates the maximum amount of the oils that could be absorbed by the StiRAs, while the latter reflects the maximum degree of oil desorption after switching the surface wettability. As one step further, it would be interesting to explore the possibility of introducing both magnetic‐responsive property and switchable surface wettability on the same absorbent. Such novel dual‐responsive absorbents could thereby benefit from the magnetic‐driven collection, as well as being able to retrieve oil resources by switching their surface wettability. To the best of our knowledge, further investigations are still needed in this field of study.

##### pH‐Responsive Absorbents

**Figure 15:**
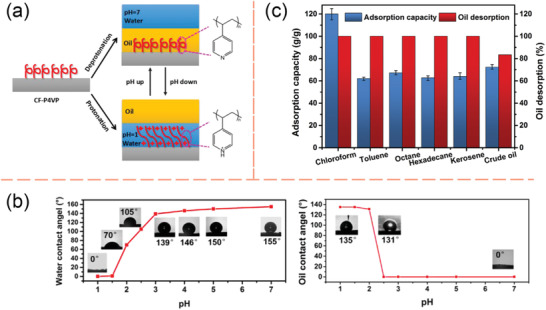
a) Schematic diagram of P4VP‐coated CF surface in water environment at neutral pH (hydrophobic/oleophilic) and low pH (hydrophilic/oleophobic). b) Water contact angle and under‐water oil contact angle of the CF–P4VP surface at different pH conditions. c) Adsorption capacity of CF‐P4VP to various types of oils at pH = 7 (blue bars) and their desorption efficiency in water of pH = 1 (red bars). Reproduced with permission.^[^ ^202^ ^]^ Copyright 2019, Elsevier.

pH‐responsive absorbents (pH‐RAs) are one of the most studied StiRAs due to their quick response and easy applicability. They are typically designed with absorbing properties at neutral/mild acidic pH (seawater condition) and switch to their desorbing form in strongly acidic solutions. The reason for switching to the low pH region is to avoid awakening the natural surfactants that are formed in basic conditions.^[^ ^46^, ^120^ ^]^ Although these natural surfactants can be helpful in releasing of oil, they may also foul the absorbent surfaces due to specific adsorption, hindering the switchability and recyclability of pH‐RAs. One representative example of pH‐RAs was given by Xu et al., who coated a layer of pH‐responsive polymer, poly(4‐vinylpyridine) (P4VP), onto carbon foam (CF).^[^ ^202^ ^]^ The switchable surface wettability was introduced by the protonation/deprotonation of the pyridyl groups in P4VP (**Figure** **15**a). When pH = 7, P4VP chains are in their deprotonation form. They exhibit hydrophobic/oleophilic properties, which favor the oil absorption process. When the solution pH decreases, the pyridyl groups are protonated into their ionic form, thus showing hydrophilic/oleophobic properties as suggested by the low WCA and high under‐water oil contact angle (UWOCA) values (Figure 15b). Consequently, the oil‐soaked CF‐P4VP could release almost 100% of hydrocarbons when being immersed into acidic solutions of pH = 1, except for the case of crude oil (83%) (Figure 15c). The authors^[^ ^202^ ^]^ claimed that the relatively higher viscosity of crude oil was the major issue causing less efficient desorption.

##### Thermo‐Responsive Absorbents

**Figure 16:**
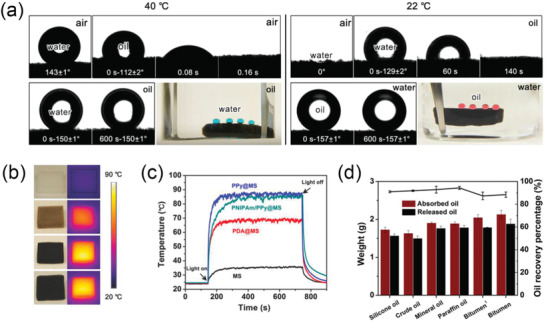
a) Surface wettability of the MS‐PNIPAm/PPy at temperature of 4 °C (left) and 2 °C (right). b) Digital images (left) and IR thermal images (right) of original MS, MS‐PDA, MS‐PPy, and MS‐PNIPAm/PPy (from top to bottom). c) Time‐dependent top surface temperature changes in response to 1000 W m^−2^ light irradiation. d) Oil absorption capacity and desorption efficiency of the MS‐PNIPAm/PPy. Reproduced with permission.^[^ ^205^ ^]^ Copyright 2018, Wiley‐VCH.

Functional surfaces with thermo‐switchable wettability are another attractive option for developing novel thermo‐responsive absorbents (thermo‐RAs). As mentioned in Section 2.3, thermo‐responsive polymers exhibit a coil‐globule transition when bypassing a critical temperature,^[^ ^48^ ^]^ which switches their water/oil affinity. Therefore, thermo‐RAs could easily be created by grafting thermo‐responsive polymers onto the surfaces of porous materials.^[^ ^203^, ^204^, ^205^ ^]^ If the polymer performs LCST‐type behavior (i.e., transforms from water‐soluble to water‐insoluble when the temperature rises above its LCST), the as‐generated absorbents would exhibit a decent oil‐absorbing property at high temperatures (*T* > LCST) and switch to oil‐repelling in a cold environment (*T* < LCST). Since the LCST could be much lower than the distillation temperature, the total energy investment for the whole adsorption‐desorption cycle is reduced. More recently, Wu et al. developed a light‐induced thermo‐responsive oil absorbent (MS‐PNIPAm/PPy) for the purpose of ultra‐heavy oil spill cleanup.^[^ ^205^ ^]^ The absorbent surfaces performed superhydrophobic and oil‐wetting properties at 40 °C. They could be transformed into superoleophobic and water‐wetting at 22 °C (**Figure** **16**a), which was attributed to the LCST behavior of PNIPAm chains. Each state of the surface wettability corresponds to the needs of oil absorption and desorption stages, respectively. More interestingly, the high‐temperature condition was induced by the sunlight‐responsive moiety (Polypyrrole, PPy), which could heat up the entire absorbent to as high as 84 °C under nonconcentrated sunlight irradiation (1000 W m^−2^) (Figure 16b,c). Such a localized heat generation was sufficient enough to not only maintain the absorbents in their oil‐absorbing state, but also to accelerate the absorbing process by reducing the oil viscosity.^[^ ^205^, ^208^ ^]^ On the other hand, the subsequent oil desorption process could have taken place at room temperature without sunlight. Collectively, these novel thermo‐RAs demonstrated both decent oil absorption and desorption properties, as shown in Figure 16d. Aside from the rapid development of thermo‐RAs, the major limitation of this type of material is the low thermal conductivity of their porous scaffold, which leads to a slow switching process and, usually, incomplete oil desorption.^[^ ^204^ ^]^ In order to address such issues, Lei et al. reported that cutting the thermal‐responsive absorbents into small pieces (0.5 × 0.5 × 1 cm^3^) could impact the slow heat transfer. The authors found that the oil desorption was much more rapid and effective if their thermo‐RAs were cut into small pieces (which resulted in little residual oil after 135 s) rather than using larger ones (which resulted in incomplete desorption after 6 h).

##### UV‐Responsive Absorbents

**Figure 17:**
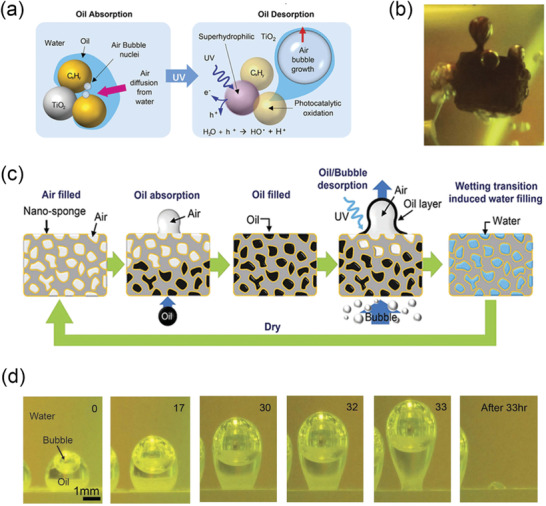
a) Mechanism of bubble growth and oil desorption with UV‐responsive TiO_2_ NPs. b) Under‐water crude oil desorption after UV irradiation with the assist of air bubbling. c) A schematic procedure of oil absorption and desorption on the UV‐responsive absorbent assisted by the air bubble flow. d) Spontaneous growth of bubbles within an oil droplet and oil/bubble release behavior on the surface of the nano‐sponge underwater with UV irradiation. Reproduced under terms of the CC‐BY license.^[^ ^9b^ ^]^ Copyright 2015, Nature Publishing Group.

Switchable wettability induced by UV irradiation has also been demonstrated to be an effective means of controlling oil absorption and desorption.^[^ ^92^, ^206^ ^]^ Kim et al. reported a UV‐responsive absorbent (UV‐RA) by mixing hydrocarbon NPs and TiO_2_ NPs.^[^ ^9b^ ^]^ The hydrophobic nature of hydrocarbon NPs enables efficient oil absorption, while the UV‐induced wettability transition of TiO_2_ NPs from mild oleophilicity to strong under‐water oleophobicity benefits the desorption of oil components (**Figure** **17**a). In an effort to maximize the desorption efficiency, air bubbles were injected to enhance the separation process (Figure 17b–d). It was demonstrated that 98 ± 1% of the oil was removed from the absorbent, leaving a water‐filed “clean” sponge. Although UV light as the switching trigger is facile and direct, it is still worth considering whether the applied UV light could reach the absorbent surfaces and efficiently switch the surface wettability. As known, oil spills composited of “black” petroleum oil are of low transparency and low UV light penetration depth,^[^ ^209^ ^]^ both of which reduce the probability of UV light reaching the absorbent surface and switching the UV‐responsive moieties. Therefore, UV‐RAs typically require a long exposure to the UV light in order to achieve the desired degree of switching. For example, the UV‐RA produced by Kim et al. requires a 16 h UV irradiation to complete the oil desorption.^[^ ^9b^ ^]^ Moreover, there are also reports addressing that the toxicity of oil spills might be enhanced by the UV irradiation, which might cause further environmental concerns.^[^ ^4^, ^210^ ^]^

In addition, the air bubble‐assisted desorption process is heavily relied on the spreading of oil layer on air bubbles. It is most desirable to have the air bubbles fully engulfed by the oils to maximize the desorption efficiency. For the UV‐RAs reported by Kim et al., the authors claimed that the calculated spreading coefficient (S) of the heavy oil on air bubble has a positive value (+ 3.8 mN m^−1^),^[^ ^9b^ ^]^ which should lead to complete oil engulfment. However, experimental results only shows the partially wetting morphology of the oil layer on the air bubble (Figure 17d). It is most likely that the adhesion force between the heavy oil and the absorbent surface provides an extra drag force on the body of the oil droplet and changes wetting configuration at the oil‐water‐air three phase contact line. Nevertheless, it is possible to modulate the spreading coefficient (*S*) by tuning the IFTs of oil‐water (*γ*_ow_), air‐water (*γ*_aw_), and air‐oil (*γ*_ao_) interefaces in an effort to promote oil engulfment. For example, Zhang et al. reported that the addition of appropriate colloidal particles can change the wetting configurations significantly.^[^ ^211^ ^]^ Clearly, there are many research opportunities on the fundamental understandings of oil engulfment phenomenon, especially for the systems involving high viscosity oils, which is essential not only for the improvement of oil desorption efficiency on novel UV‐RAs, but also for many industrial applications, such as flotation‐assisted oil recovery, contrast‐enhanced ultrasonography, and defoaming.

## 6 Conclusions and Outlook

**Scheme 6:**
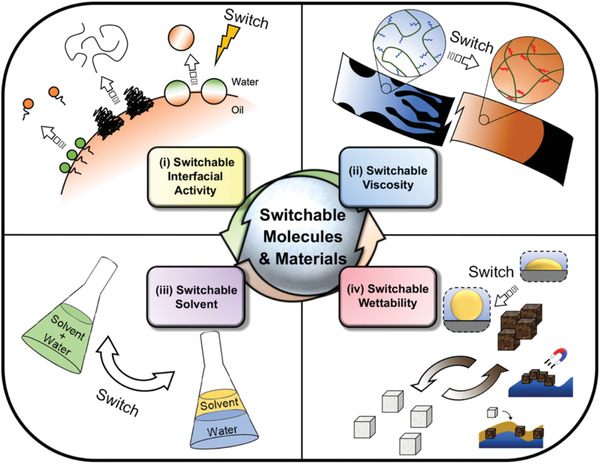
Strategies of using switchable molecules and materials for oil recovery and oily waste cleanup applications: i) Switchable interfacial‐active materials (e.g., surfactants, polymeric surfactants, and interfacially active particles) can be utilized to introduce controlled emulsification and demulsification via external stimulus; ii) Polymer solutions with switchable viscosity are applied in the enhanced oil recovery (EOR) to reduce viscous fingering effect while remaining good pumping efficiency; iii) Switchable solvents could greatly facilitate the recovery of hydrocarbons from reservoir or contaminated soil in their non‐polar form, while being recycled conveniently by switching into their polar form; iv) Novel absorbents are most desirable to be designed with superoleohyphilic/superhydrophoblic surfaces for excellent oil absorption capacity, with magnetic‐responsive property for convenient after‐cleaning collection, as well as with switchable surface wettability for facile oil desorption and robust recyclability.

In this review paper, we discussed the considerations in designing advanced switchable molecules and materials for both oil recovery and oily waste cleanup applications. **Scheme** **6** summarizes the four major strategies that are typically involved in these specific applications, including switchable interfacial activity, switchable viscosity, switchable solvent, and switchable wettability.

Responsive materials with switchable interfacial activity, including switchable surfactants, switchable polymeric surfactants, and switchable particles, have been widely investigated for the purpose of both oil recovery and oily waste cleanup. In general, introducing interfacial‐active materials can facilitate the oil‐solid separation and oil emulsification in water, while the ability to switch off their interfacial activity could greatly facilitate the subsequent stage of oil‐water separation. The biggest challenge for such a group of switchable materials is their irreversible adsorption on solid surfaces, which makes them less effective at reducing oil‐water interfacial tension, as well as hinders their switchability. Therefore, it is essential to select the switchable surfactants with appropriate charges at their ionic state, e.g., cationic surfactants for carbonate reservoirs, such that the adsorption of surfactants on solid surfaces is less significant. In some other applications, vapor‐phase processes are considered to be an alternative way of tuning surface adsorption.^[^ ^212^ ^]^ However, one has to admit that more pioneering efforts would be needed for the proof‐of‐concept before implementing such exploratory concepts.

Switchable materials with tunable viscosity are mostly applied in enhanced oil recovery applications. Switchable polymer solutions or self‐assembly solutions are designed with good fluidity and injectability for improved pumping efficiency, while they ideally switch to high‐viscous fluids near the oil‐rich region in the reservoir in order to reduce the viscous fingering effect and increase oil recovery. The switching behavior could be triggered by certain internal variations (temperature) or by an external stimulus (pH, CO_2_). Although there is an increasing number of reports on these switchable viscosifiers from laboratory‐scale demonstrations to pilot‐scale implementation, the current enhancement in oil recovery is currently not economically favored to compensate for the cost of using switchable materials. In addition, the switching behavior requires further understanding of reservoir conditions, including high temperatures, high salinity, and high porosity.

Switchable solvents have recently attracted considerable attentions as the next generation of green solvents. Switchable hydrophilicity solvents (SHSs) that can switch from oil‐soluble to oil‐insoluble reversibly are the most studied switchable solvents for the oil recovery applications. SHSs have been investigated for solvent extraction and solvent‐assisted ambient aqueous hybrid extraction processes, both of which show promising improvement in the oil recovery ratio. However, industry and environmental regulators still consider the solvent loss of SHSs unacceptable because of their incomplete switching behaviors. As for the other members of switchable solvents, there are also a few reports about developing novel switchable waters for the oil sands extraction, whereas switchable polarity solvents are considered to be unsuitable for most oil recovery and oily waste cleanup applications.

Novel absorbents with switchable surface wettability have been extensively studied in recent years for oil spill cleanup. MagRAs are designed to conveniently collect oil‐soaked absorbents with the guidance of external magnetic fields. In the meantime, StiRAs are being investigated to facilitate oil desorption from as‐generated oil‐soaked absorbents, which can recover the valuable petroleum hydrocarbons and recycle the clean absorbents. There are two major obstacles to developing responsive absorbents: the range and strength of the magnetic responsiveness, especially in the marine environment; and the low desorption efficiency for high viscous oil from the absorbents after the switching behaviors are activated.

Future development of switchable molecules and materials for oil recovery and oily waste cleanup application could be assigned into two major directions: the fundamental understanding of switching behaviors, and the development of novel materials with better performance and feasibility.

For the first aspect, we believe that there are emerging needs to extend the current understandings of switching behavior. Currently, switchable materials are mostly compared by their static properties before and after the switch. However, it has to be highlighted that the switching process is a dynamic process. Therefore, a comprehensive understanding of the switching behavior should not only consider the static differences between switching‐on/off states, but also involve how fast the switching would be. From the application perspectives, faster switching behavior is most likely to benefit the process, since the transition region between the switching‐on/off states of a switchable material is commonly the inefficient zone in operations. Therefore, the dynamics of switching behaviors are a field of critical importance, but, to the best of our knowledge, is less explored. Besides, recent studies showed that the switching behavior could also be influenced by the interfacial environments (e.g., oil‐water interface). For example, switchable surfactants have been widely reported to have different *pK*_a_ values (switching pH) at the interface and in the bulk solution.^[^ ^10^, ^213^ ^]^ Unfortunately, most *pK*_a_ values reported in literature correspond to the solution switching behavior, whereas the interfacial switching behavior is believed to be more relevant to the application scenarios (switch at the interface). Moreover, there is little information regarding the switching behaviors in complex environments (e.g., porous media, oil‐water‐solid mixtures). Such research would provide comprehensive information to assess the way that switchable molecules/materials perform in actual applications.

On the other hand, it is also challenging to transfer the cutting‐edge development of switchable materials into these large‐scale applications. For example, there has been a rapid development of responsive behaviors that could be driven by interfacial energy. In these cases, the stimuli are presented immediately and explicitly at the interface. Such a concept has drawn much attention in the fields of self‐healing materials and molecular recognition technology,^[^ ^214^ ^]^ and might be promising in the development of novel absorbent, membrane, and pipeline materials.

In summary, switchable materials have been demonstrated to have significant advantages over conventional materials in various areas, while numerous research opportunities are presented in resolution of the challenges and limitations that hinder the vast industrial application of these advanced materials. The successful applications of switchable materials require a comprehensive understanding of material properties, switching behaviors, as well as process conditions and limitation. All in all, further research on the fundamental knowledge are the key to developing next‐generation switchable materials for the relevant applications.

## Conflict of Interest

The authors declare no conflict of interest.
